# The role of VdSti1 in Verticillium dahliae: insights into pathogenicity and stress responses

**DOI:** 10.3389/fmicb.2024.1377713

**Published:** 2024-04-04

**Authors:** Yutao Wu, Jinglong Zhou, Feng Wei, Yalin Zhang, Lihong Zhao, Zili Feng, Hongjie Feng

**Affiliations:** ^1^Zhengzhou Research Base, State Key Laboratory of Cotton Biology, School of Agricultural Sciences, Zhengzhou University, Zhengzhou, China; ^2^National Nanfan Research Institute (Sanya), Chinese Academy of Agricultural Sciences, Sanya, China; ^3^State Key Laboratory of Cotton Biology, Institute of Cotton Research of Chinese Academy of Agricultural Sciences, Anyang, China

**Keywords:** Sti1/Hop, Verticillium dahliae, microsclerotia, stress response, pathogenicity

## Abstract

Sti1/Hop, a stress-induced co-chaperone protein, serves as a crucial link between Hsp70 and Hsp90 during cellular stress responses. Despite its importance in stress defense mechanisms, the biological role of Sti1 in Verticillium dahliae, a destructive fungal pathogen, remains largely unexplored. This study focused on identifying and characterizing Sti1 homologues in *V. dahliae* by comparing them to those found in *Saccharomyces cerevisiae*. The results indicated that the VdSti1-deficient mutant displayed increased sensitivity to drugs targeting the ergosterol synthesis pathway, leading to a notable inhibition of ergosterol biosynthesis. Moreover, the mutant exhibited reduced production of microsclerotia and melanin, accompanied by decreased expression of microsclerotia and melanin-related genes VDH1, Vayg1, and VaflM. Additionally, the mutant’s conidia showed more severe damage under heat shock conditions and displayed growth defects under various stressors such as temperature, SDS, and CR stress, as well as increased sensitivity to H2O2, while osmotic stress did not impact its growth. Importantly, the VdSti1-deficient mutant demonstrated significantly diminished pathogenicity compared to the wild-type strain. This study sheds light on the functional conservation and divergence of Sti1 homologues in fungal biology and underscores the critical role of VdSti1 in microsclerotia development, stress response, and pathogenicity of *V. dahliae*.

## 1 Introduction

The stress-induced auxiliary chaperone protein Sti1/Hop, also known as Hsp histone, is an Hsp90 auxiliary chaperone protein that is widely distributed in fungi and sharing homology with the mammalian Hsp90 auxiliary protein Hop (Song et al., 2009; Reidy et al., 2018). Sti1/Hop was initially identified in yeast as a mediator of the heat shock response for certain Hsp70 genes (Nicolet and Craig, 1989). It acts as an adaptor protein that binds to both Hsp70 and Hsp90, facilitating the transfer of client proteins from Hsp70 to Hsp90 (Young et al., 2001). Subsequently, Sti1/Hop was cloned and characterized as a molecular chaperone protein in mice and other mammals (Blatch et al., 1997). Extensive investigations have been conducted to study the function of Sti1 in various species, including yeast, plants, and mammals. As an Hsp90 accessory chaperone, Sti1 plays a crucial role in regulating various cellular processes and biological functions (Schwarz et al., 2023). In mice, overexpression worsens Aβ accumulation and AD plaque formation (Lackie et al., 2020). Hypomorphic mutant has less synuclein inclusions and phenotypic effects in synucleinoverexpressing strain (Lackie et al., 2022). In concert with Hsp90, Hop/Sti1 modulates TDP-43 misfolding, aggregation, and toxicity in yeast, mammalian cells and embryos (Lin et al., 2021). These studies have illuminated the diverse roles of Sti1 in different organisms and provided valuable insights into its contribution to cellular homeostasis, stress response, and protein regulation (Gu et al., 2016; da Fonseca et al., 2021).

Fungal plant pathogens present a significant threat to global food security and biofuel production. Soil-borne pathogens such as *Verticillium* and *Fusarium* fungi can cause destructive vascular wilt diseases in plants (Berg et al., 2000; Ruiz-Roldán et al., 2008). Verticillium wilt is characterized by fungal hyphae colonizing and clogging the plant’s vascular tissues, leading to wilting and eventual death of affected leaves (Klosterman et al., 2009; Prieto et al., 2009). The presence of micronuclei in necrotic plant tissues can promote the survival of pathogenic bacteria in the soil, creating a significant challenge for disease management in agriculture (Klimes et al., 2015). *V. dahliae* demonstrates notable variability and co-evolutionary abilities with its host, and its pathogenic mechanism is intricate (Depotter et al., 2019; Lv et al., 2022; Zhang et al., 2022). The specific interaction between the pathogen and the host remains incompletely understood. Nonetheless, unraveling the molecular pathogenic mechanism of *V. dahliae* holds promise for effectively combating yellow wilt disease in cotton.

The majority of research on the Sti1 protein has predominantly focused on higher-order eukaryotes, such as yeasts and model plants, with comparatively less attention given to understanding its functions and mechanisms in fungal pathogens. While limited, some studies on Sti1 in fungal pathogens, like *Candida albicans*, have begun to reveal its involvement in virulence traits like biofilm formation, filamentation, and adhesion to host tissues (Ni et al., 2004). This study aims to investigate the role of Sti1 in *V. dahliae*, exploring its function and regulation of relevant pathways to enhance our understanding of its role and underlying mechanisms in pathogenicity among related fungal pathogens. By investigating how Sti1 influences pathogenicity in *V. dahliae*, valuable insights into the intricate relationship between the fungal pathogen and its host plant are expected to be uncovered. This knowledge could aid in developing innovative strategies for precise disease management and enhancing crop production.

## 2 Materials and methods

### 2.1 Growth conditions for plants and fungi

The study employed the highly pathogenic Vd080 strain of *V. dahliae* (Zhang et al., 2015; Zhu et al., 2021). The fungus was cultured on potato dextrose agar medium and incubated in a dark environment at 25°C. To collect conidia, fungal plugs were grown in liquid Czapek-Dox broth and shaken at 200 rpm for 5 days at 25°C. The virulence assessment was conducted on cotton plants of the Jimian-11 variety, which is known to be susceptible. The cotton plants were cultivated in a greenhouse under controlled conditions with a 16 h light/8 h dark cycle at 25°C.

### 2.2 Generation of knockout and complementation mutant strains

To generate the knockout mutant strain, upstream and downstream flanking sequences of the target gene were amplified from the genomic DNA of *V. dahliae*. An hygromycin resistance cassette (HPH) was amplified from the B303 vector and cloned into the B303 vector (Lv et al., 2023). The constructed vector was then transformed into *Agrobacterium tumefaciens* AGL1, and the knockout mutant was generated through *Agrobacterium*-mediated transformation (ATMT) (Wang et al., 2016a). For the construction of the complementation vector, a 1.5 kb upstream region of the genomic region of Vdsti1 was amplified and cloned into the pCAM-BIA1302 vector. The constructed vector was transformed into *A. tumefaciens* AGL1. The complementation strain was then generated through ATMT (Paz et al., 2011; Li et al., 2012; Zhou et al., 2013). NCBI’s Primer Blast tool was used for primer design.^1^ DNAMAN was used to align multiple protein sequences. The primers used in this experiment are listed in Supplementary Table S1.

### 2.3 Determination of ergosterol

The spore suspension should be inoculated into Potato Dextrose Broth (PDB) culture medium and incubated at 25°C for 3 days. Follow the specific extraction method for ergosterol (Liu et al., 2013). The experiment should be conducted using a Waters 2,695 High Performance Liquid Chromatography system equipped with a Waters 2,996 UV detector. Ergosterol is separated on a Waters C18 column (5 μm, 4.6 mm × 250 mm) at 30°C, using 100% methanol (chromatographic grade) as the mobile phase. The detection wavelength is set at 282 nm (Dushkov et al., 2023; Fernandes et al., 2023; Manita et al., 2024). The standard for ergosterol is purchased from Shanghai YuanYue Biotechnology Co., Ltd. The experiment is repeated three times.

### 2.4 Stress response

To determine temperature stress, activate all strains on PDA mediumat 16°C, 25°C, and 30°C for 14 days. Then, cultivate spores in a chaotrophic medium to prepare the spore suspension. Subject the spore suspensions of each strain to a heat shock at 42°C for 30 min. Observe the morphology of conidia under an electron scanning microscope. Afterward, subject the spore suspensions of each strain to a heat shock at 50°C for 30 min. Evenly spread the spore suspensions onto PDA plates and incubate them in a 25°C incubator. Calculate the spore viability after 3 days of incubation (Tang et al., 2017).

To determine abiotic stress, 1 mol/L KCl, 1 mol/L NaCl, 1 mol/L Sorbitol, 0.004% SDS and 0.02% CR were added to PDA medium (Lv et al., 2022; Liu et al., 2023). The control group was grown on normal PDA medium.

For oxidative stress determination, 100 μL of spore suspension was spread onto PDA plates, and filter paper discs soaked with 5 μL of 15% hydrogen peroxide (H_2_O_2_) was placed in the center of each plate. Measure the diameter of the inhibition zones after 3 days of growth (Tang et al., 2017; Rehman et al., 2018; Tang et al., 2020). Ensure to repeat all experiments three times. The primers utilized in this experiment are available in Supplementary Table S2.

### 2.5 Observation of microsclerotia and analysis of related gene expression

To study the production of microsclerotia, fresh spore suspensions were incubated flat on solid basal medium (BM) containing cellulose membranes for 15 days. Microsclerotia were then observed using a stereomicroscope (Xiong et al., 2014). The experiment was repeated three times.

For the investigation of the impact of VdSti1 knockout on the expression of genes related to other microsclerotia and melanin formation, the mycelium from different strains was collected. Total RNA was extracted from each mycelium using an RNA extraction kit (YPHBio, Tianjin, China). The extracted RNA was then used for cDNA synthesis using the HiScript II QRT SuperMix (+gDNAwiaper) kit (Vazyme). The obtained cDNA was subjected to qPCR analysis to determine the relative expression levels of genes *VDH1*, *Vayg1*, and *VaflM* (Klimes and Dobinson, 2006; Klimes et al., 2008; Fan et al., 2017; Li et al., 2020). The primers used in this experiment are listed in Supplementary Table S3.

### 2.6 Drug sensitivity test

In this experiment, the sensitivity of *Verticillium dahliae* to the amine-type drug Tridemorph and the non-amine type drugs Triadimefon and Tebuconazole was evaluated. The EC50, which represents the half-effective concentration of each drug against *V. dahliae*, was determined following a previously established method (Gu et al., 2016). Different strains of *V. dahliae* were inoculated onto PDA medium supplemented with the respective drugs. After 14 days of incubation, the growth diameter of colonies was measured to assess the inhibitory effect of each drug on fungal growth. Each test was repeated at least three times.

### 2.7 Pathogenicity assay

To prepare spore suspensions of various strains, adjust the concentration to 1 × 10^7^ CFU/mL. Immerse water-cultured cotton seedlings (4 weeks old) in the spore suspension for 2 min. Then, transfer the seedlings back into the nutrient soil and place them in a cotton greenhouse for planting. After 21 days of inoculation, record the disease index based on the previous method and take photographs to document the disease occurrence. The symptoms of the cotyledons and true leaves are graded on a scale of 0 to 4 (Gong et al., 2017; Zhang et al., 2023). Using a surgical knife, make a longitudinal cut at the same location on the stems and roots of cotton plants harvested after 21 days of inoculation. Observe the extent of browning on the cotton stems under a stereomicroscope (Leica, M165 FC, Germany).

The stems were harvested 21 days after inoculation and ground to powder. Total DNA was extracted from the cotton plants using the Plant Genome Extraction Kit (Vazyme). The biomass of *V. dahliae* in the stalks was determined using the 2^−ΔΔCT^ method. The target gene for the assay was *Vdβt*, and *GhUBQ7* served as the internal reference gene. The primers used in this experiment can be found in Supplementary Table S4.

### 2.8 RNA extraction and RT-q-PCR analysis

A 1 μL conidial suspension (1 × 107 CFU/mL) of both the wild type and VdSti1 knockout mutant strains were inoculated in 200 mL of PDB and incubated on a shaker (180 rpm) at 25°C. After 5 days, the culture was filtered through four layers of clean gauze to collect hyphae. Total RNA was extracted from the respective hyphae using the RNA Extraction Kit (YPHBio, Tianjin, China). First-strand cDNA was synthesized with HiScript II QRT SuperMix for qPCR (+g DNA wiper) (Vazyme) according to the instruction. qRT-PCR was performed using ChamQ Universal SYBR qPCR Master Mix (Vazyme).s. The relative expression of each gene was determined using the 2^−∆∆Ct^ method, as previously described. All experiments were repeated three times. The primers used in RT-qPCR are listed in Supplementary Table S5.

### 2.9 Yeast dual hybrid screening for interacting proteins and validation

PGBKT7-VdSti1 was used as bait to screen interacting proteins from the yeast AD-cDNA library of *V. dahliae* (provided by our laboratory). The screened proteins were sequenced using NCBI^2^ and then cloned into pGADT7 for yeast heterozygosity verification with pGBKT7-VdSti1. Transfer the recombinant plasmids of the two interacting proteins into yeast receptive cells. Coat them in two deficient culture media (SD Leu Trp) and dilute the single colony with sterile water after growth. Then, transfer them to cells containing X-α-gal dye four deficient media (SD His Leu Trp Ade) and observe their growth after 1 week.

*Agrobacterium tumefaciens* containing plasmids VdSti1-nLUC and VdExt2-cLUC were mixed and uniformly injected in a 1:1 ratio into 4 week-old tobacco leaves. The leaves were then cultivated in a dark environment for 1 day, followed by transfer to normal cultivation conditions for one day. The fluorescence signal of the tobacco leaves was detected using a weak light cooled charge coupled device Nightshade LB985 (BERTHOLDTECHNOLOGIES, Germany). The primers used in PCR are listed in Supplementary Table S6.

## 3 Results

### 3.1 Identification of *VdSti1* in *V. dahliae*

**Figure 1:**
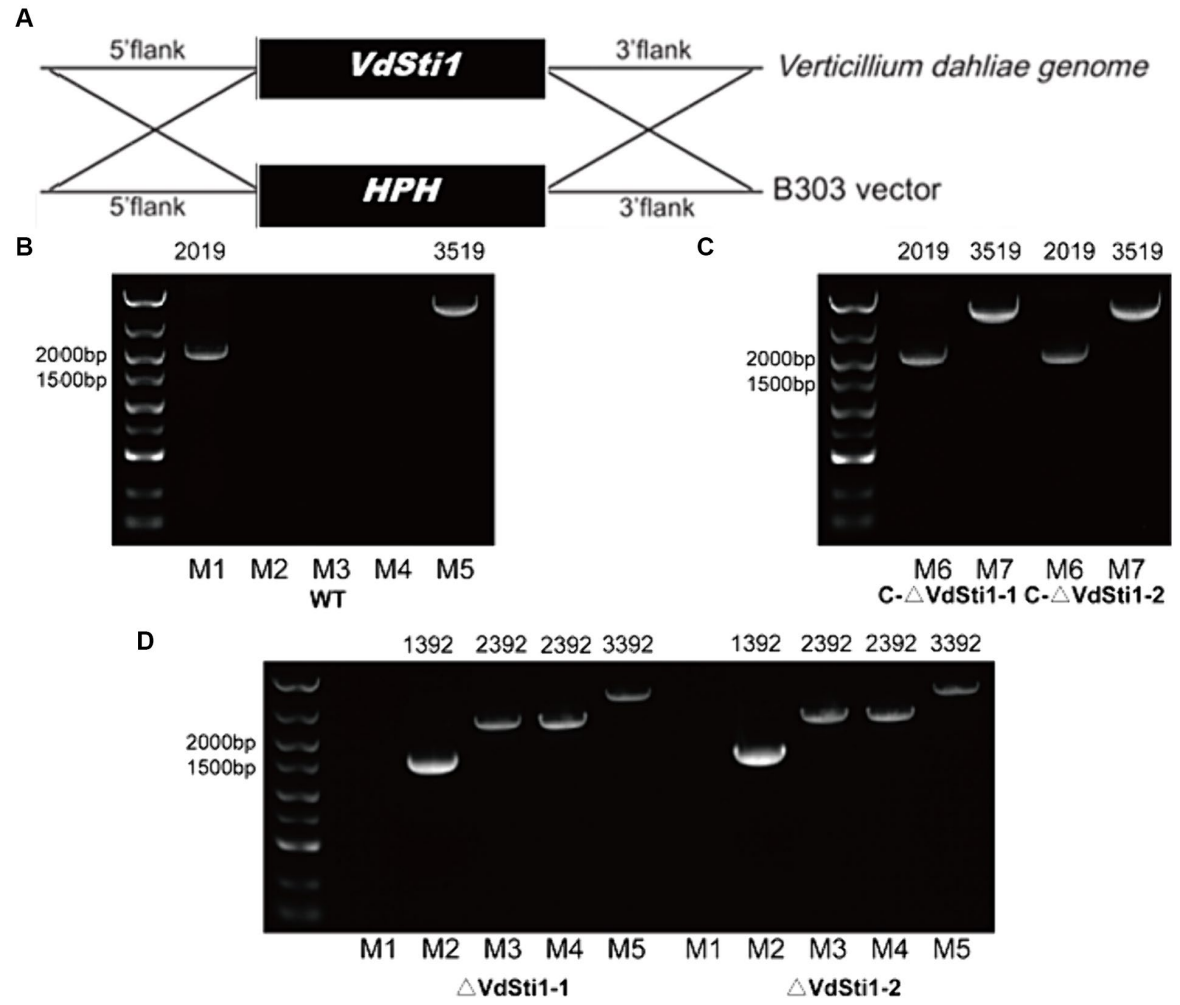
Acquisition of knock-out and complementation mutants. **(A)** Gene knockout mechanism of V. dahliae VdSti1. **(B–D)** Knockout and complementation strains were determined by PCR. (M1:*VdSti1*, M2:*HPH*, M3:UP1000 + *VdSti1*, M4:DOWN1000 + *VdSti1*, M5:UP1000 + DOWN1000 + *VdSti1*, M6:*VdSti1*, M7:UP1500 + *VdSti1*).

A homolog of Sti1/Hop, referred to as Sti1, was discovered in the genome of *V. dahliae* strain VdS.17.^3^ The VdSti1 gene (VDAG_06242) spans 1755 bp and codes for a 584 amino acid protein. A comparison of VdSti1’s amino acid sequences with other Sti1/Hop homologs using DNAMAN software indicated a high level of conservation of the Sti1/Hop protein from yeast to *V. dahliae* (Supplementary Figure S1). To investigate the specific function of the Hsp90 co-chaperone VdSti1, this study utilized homologous recombination techniques to create knockout and complementation mutants (Figure 1A). Two knockout mutants, ΔVdSti1-1 and ΔVdSti1-2, were produced, alongside two complementation mutants, C-ΔVdSti1-1 and C-ΔVdSti1-2. PCR validation confirmed the successful generation of these mutants (Figures 1B–D).

### 3.2 Deletion of *VdSti1* in *V. dahliae* leads to increased sensitivity to azoles

**Figure 2:**
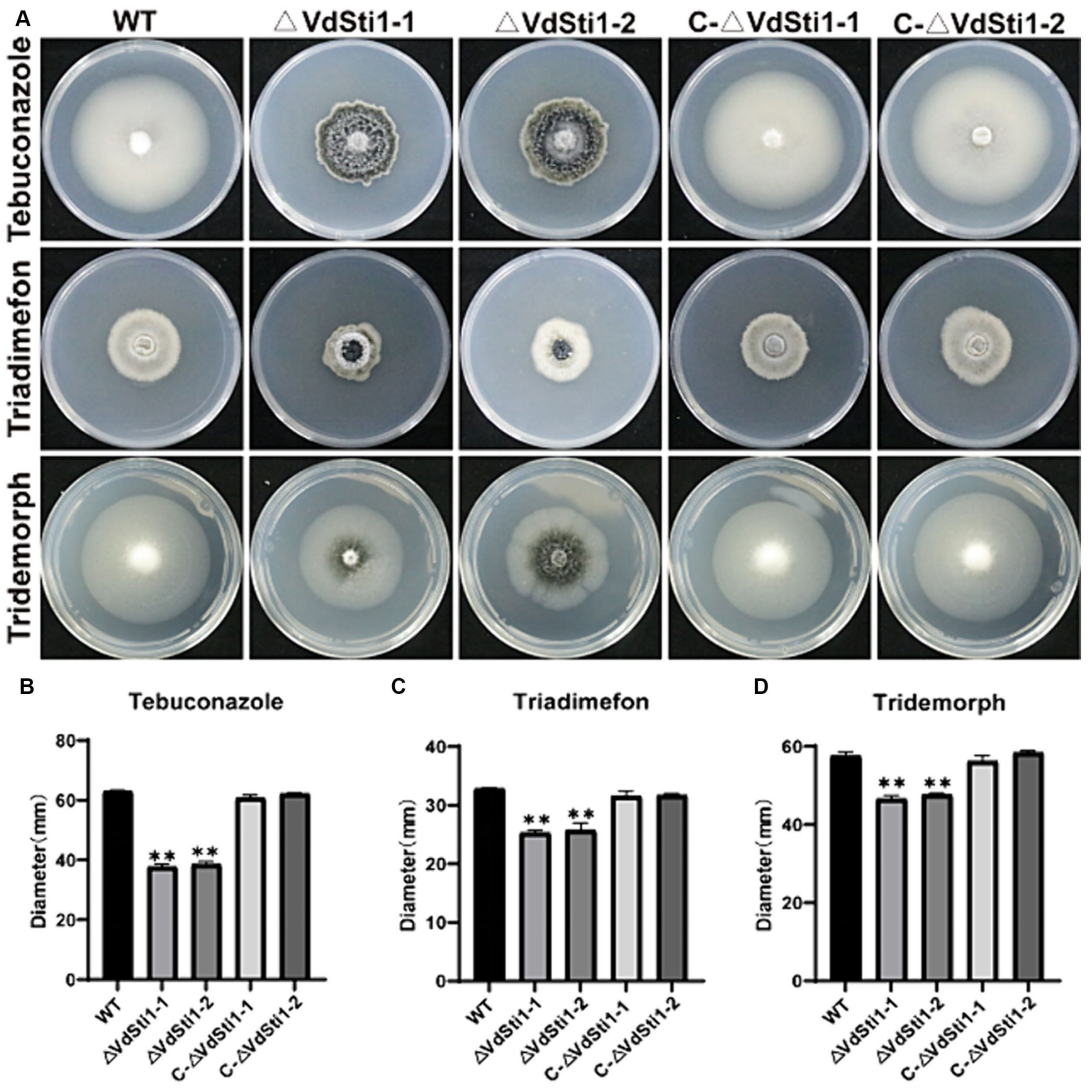
VdSti1 deletion mutants exhibit distinct responses to different fungicides. **(A)** All strains were cultured on PDA plates supplemented with 0.1 mg/L tebuconazole, 20 mg/L triadimefon and 2 mg/L tridemorph at 25°C for 14 days. Scale = 1 cm. **(B–D)** Colony diameter of all strains. Values represent means ± standard deviation of three replicates. The asterisks represent statistical differences performed by a *t*-test (*p < 0.05, **p < 0.01, and ***p < 0.001) in comparison with the wild type strains.

In order to investigate the role of Hsp90 co-chaperones in the stress response induced by amines and azoles, the half effective concentration (EC50) of each drug for inhibiting the growth of *V. dahliae*. Results showed EC50 concentrations of 0.1 mg/L for tebuconazole, 20 mg/L for triadimefon, and 2 mg/L for tridemorph, compared to the control group (Supplementary Figure S2). Notably, the growth inhibition of the VdSti1 knockout mutant was more significant than that of the wild type and complementary mutant when exposed to these drugs. Tebuconazole exhibited the strongest inhibitory effect on the knockout mutant among the tested drugs. These results strongly indicate that the Hsp90 co-chaperone VdSti1 plays a vital role in the stress response of *V. dahliae* induced by azoles (Figures 2A–D). The heightened sensitivity of the VdSti1 knockout mutant to these drugs underscores the importance of VdSti1 in modulating the stress response of *V. dahliae*.

### 3.3 *VdSti1*, in cooperation with Hsp90, regulates ergosterol production in *V. dahliae*

**Figure 3:**
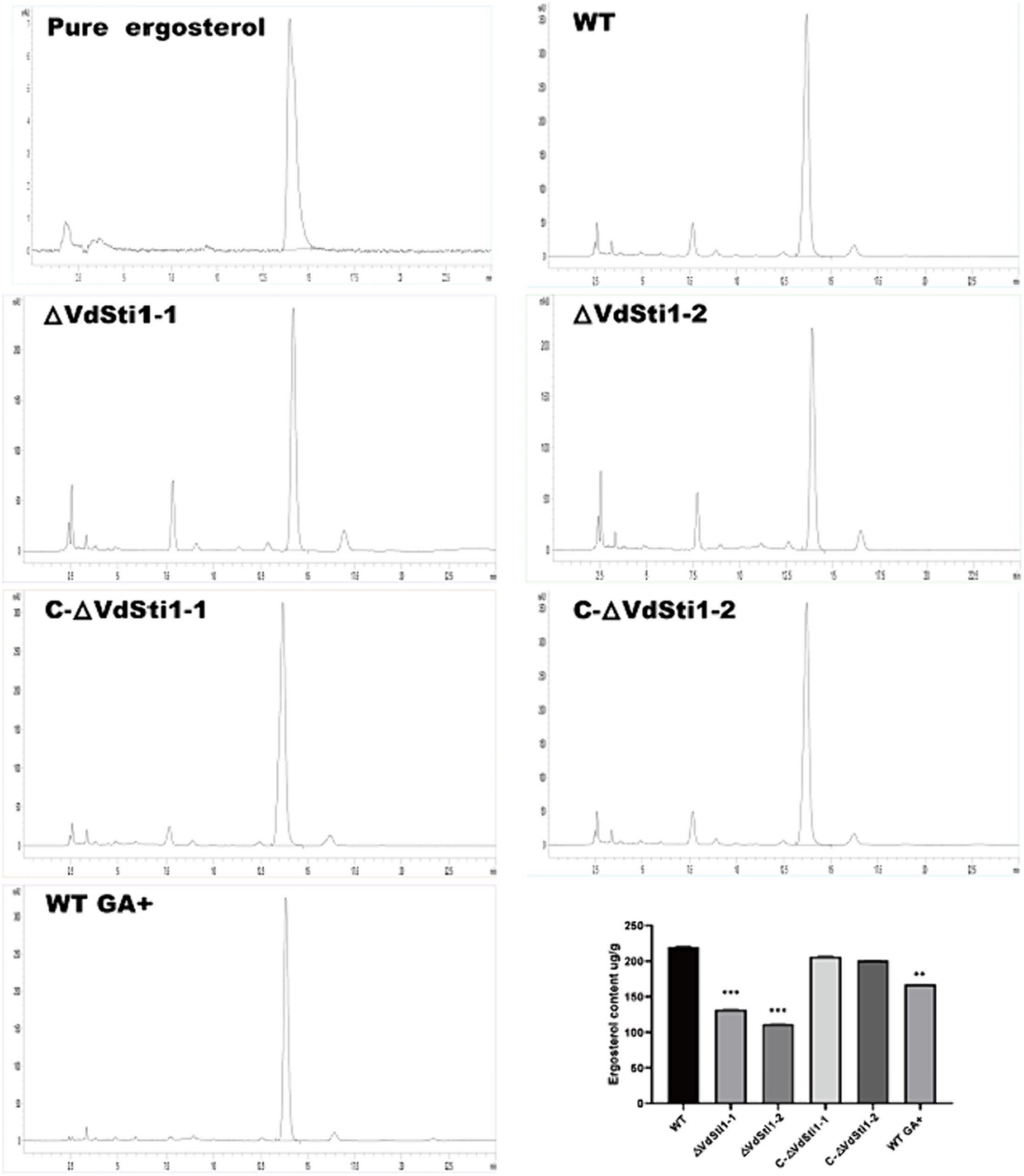
Decrease of ergosterol biosynthesis in the *VdSti1* knockout mutant. The ergosterol content in wild type, ΔVdSti1 and C-ΔVdSti1 strains were determined by high-performance liquid chromatography (HPLC). Commercial standard of ergosterol was used as the control. The ergosterol content of the wild type, the wild type (5 mg/L GA+), ΔVdSti1 and C-ΔVdSti1 strains. Values represent means ± standard deviation of three replicates. The asterisks represent statistical differences performed by a t test in comparison with the wild-type strains (*p < 0.05, **p < 0.01, and ***p < 0.001).

High-performance liquid chromatography (HPLC) analysis of ergosterol extracted from various hyphae strains, including wild-type, ΔVdSti1-1, ΔVdSti1-2, C-ΔVdSti1-1, and C-ΔVdSti1-2, showed a distinct ergosterol-specific absorption peak with a retention time of 13.7 min across all strains. Notably, the ergosterol content in the ΔVdSti1-1 and ΔVdSti1-2 mutants was notably lower than in the wild type and complementation mutants. Intriguingly, the introduction of 5 mg/L of geldanamycin, an Hsp90 inhibitor, to the wild-type strain led to reduced ergosterol production compared to the control. Despite this decrease, the wild type still exhibited higher ergosterol levels than the knockout mutants (Figure 3). These results indicate a potential crucial role for VdSti1 in the ergosterol synthesis pathway, potentially interacting with Hsp90. The decrease in ergosterol production in the knockout mutants and with geldanamycin treatment in the wild type further supports the involvement of VdSti1 and Hsp90 in the regulation of this essential fungal lipid.

### 3.4 *VdSti1* responds to temperature stress, cell wall stress, and cell membrane stress in *V. dahliae*

**Figure 4:**
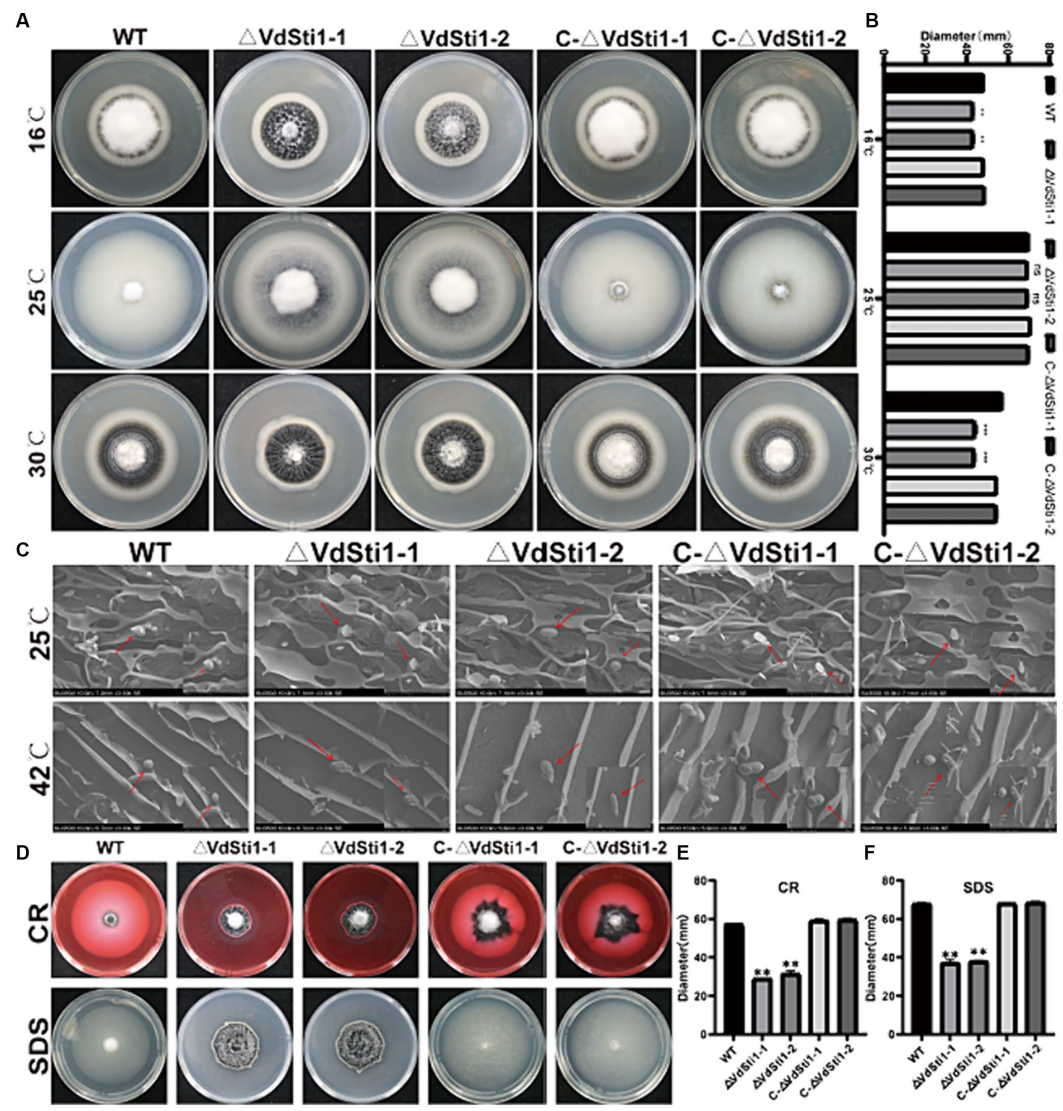
VdSti1 responds to temperature stress, cell wall stress, and cell membrane stress in *V. dahliae*. **(A)** Colony morphology of all strains cultured on PDA plates at 16°C, 25°C, 30°C in dark for 14 days. Scale = 1 cm. **(B)** Colony diameter of all strains. Values represent means ± standard deviation of three replicates. The asterisks represent statistical differences performed by a *t*-test (ns, *p* < 0.01, *p < 0.05, **p < 0.01, ***p < 0.001) in comparison with the wild type strains. **(C)** Morphology of conidia of all strains under scanning electron microscope. Scale = 4 μm. **(D)** All strains were cultured on PDA plates supplemented 0.02 %CR and 0.002% SDS at 25°C for 14 days. Scale = 1 cm. **(E,F)** Colony diameter of all strains. Values represent means ± standard deviation of three replicates. The asterisks represent statistical differences performed by a *t*-test (ns, *p* < 0.01, *p < 0.05, **p < 0.01, and ***p < 0.001) in comparison with the wild type strains.

Previous studies have shown that gene expression of the Hsp90 co-chaperone Sti1/Hop is induced under heat stress conditions in yeast and soybean. Furthermore, research on Hsp90 in *Paracocidioides brasiliensis* has revealed its role in inhibiting reactive oxygen species (ROS) production and promoting antioxidant defenses during heat-induced stress (Matos et al., 2013). To investigate the involvement of VdSti1 in stress response, the growth rate of the VdSti1 knockout mutant was evaluated at different temperatures. The results indicated that at 25°C, there were no significant differences in growth rate and conidial morphology between the VdSti1 knockout mutant strain and the wild-type or complementation mutant strains (Figures 4A–C). However, at both low temperatures (16°C) and high temperatures (30°C), the growth rate of the VdSti1 knockout mutant strain was notably lower compared to the wild type and complementation mutants (Figures 4A,B). Moreover, when exposed to high-temperature stress (42°C), the VdSti1 knockout mutant displayed conidial crumpling (Figure 4C). Additionally, the knockout mutant showed compromised conidial germination at 50°C following high-temperature treatment (Supplementary Figure S3).

To evaluate cell wall and cell membrane integrity, VdSti1 knockout mutant, wild type, and complementation mutant strains were grown on plates containing CR and SDS. Results showed that the VdSti1 knockout mutant exhibited slower growth compared to the wild type under CR and SDS stress. However, the complementation mutant displayed similar sensitivity to CR and SDS as the wild type strain (Figures 4D–F). Furthermore, to assess the response of the VdSti1 knockout mutant to osmotic stress, all strains were cultured on plates supplemented with KCl, NaCl, and Sorbitol. The growth rate of the VdSti1 knockout mutant under osmotic stress resembled that of the wild type and complementation mutants (Supplementary Figure S4). These results suggest that the deletion of VdSti1 in *V. dahliae* leads to temperature sensitivity and impairment in fungal cell wall and cell membrane integrity.

### 3.5 The deletion of *VdSti1* in *V. dahliae* resulted in heightened sensitivity to oxidative stress induced by H_2_O_2_

**Figure 5:**
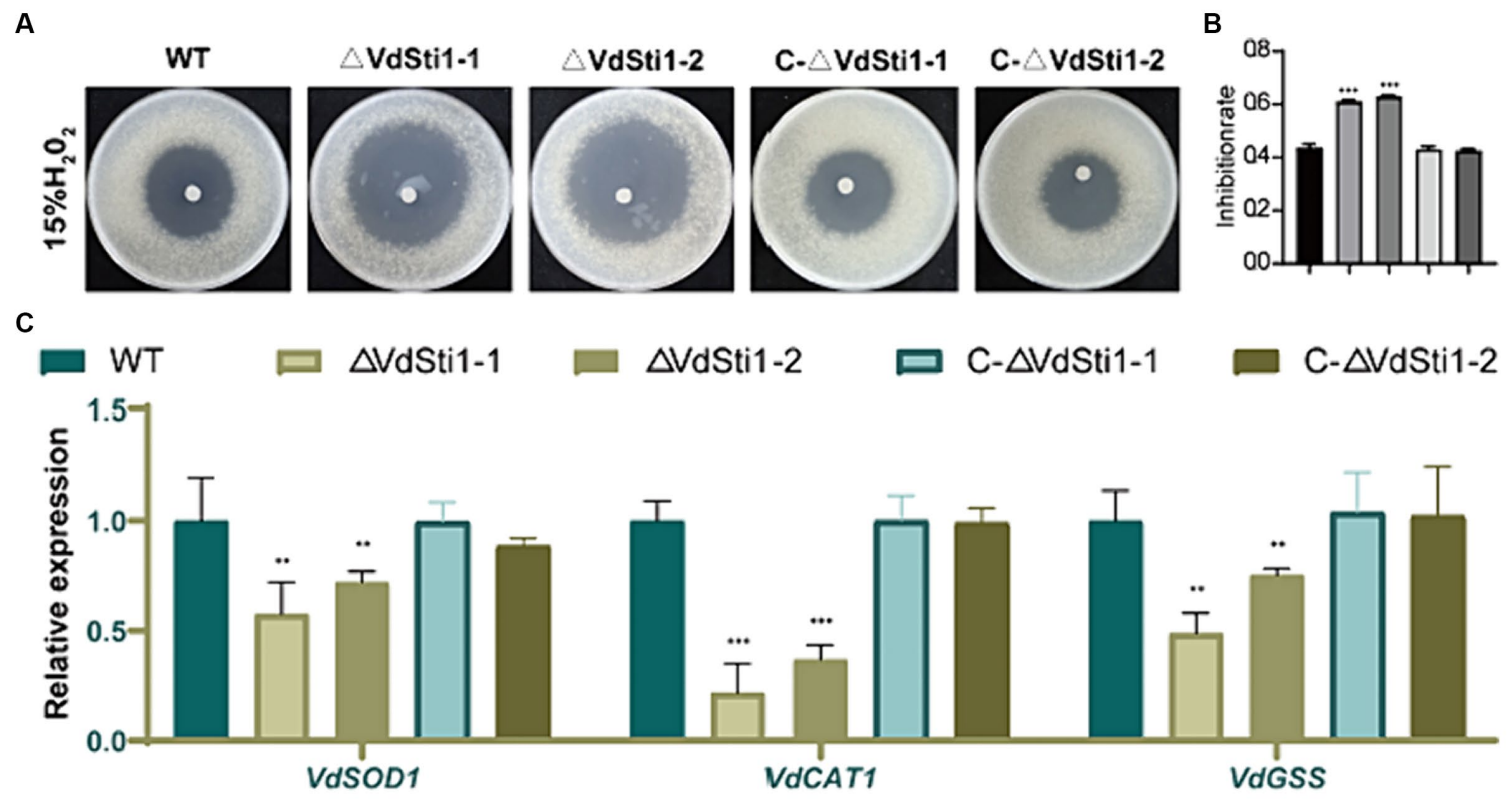
VdSti1 contributes to the oxidative stress response. **(A)** The ΔVdSti1 mutants were compared with the wild type and the C-ΔVdSti1 strain. Equal conidial suspension (1.5 mL, 1*10^10^ spores/mL) of each strain was added on PDA plates. Sterile filter paper disks with 5 mm diameters were placed in the center of the plates, and 10 μL of 15% H2O2 were added to each paper disk, respectively. The plates were incubated at 25°C for 4 days. Scale = 1 cm. **(B)** Zones of growth inhibition in **(A)** were quantified. Error bars represent standard deviation. The asterisks represent statistical differences performed by a *t*-test (ns, *p* < 0.01,*p < 0.05, **p < 0.01, ***p < 0.001) in comparison with the wild type strains. **(C)** Downregulation of genes related to peroxidase in the ΔVdSti1 mutant. Relative expression levels of three genes VdSOD1 (VDAG_08724), VdCAT1 (VDAG_03661) and VdGSS1 (VDAG_06340), which encode peroxidases, were determined by qRT-PCR using the RNA from mycelium treated with 1 mM H2O2 for 30 min. Error bars represent standard deviation.

To evaluate the sensitivity of the ΔVdSti1 mutant to H2O2, an H2O2 sensitivity assay was performed. The results demonstrated that the ΔVdSti1 mutant displayed significantly higher sensitivity to H2O2 compared to the wild-type and complementation mutant strains. The zone of inhibition caused by H2O2 was notably larger for the ΔVdSti1 mutant (Figures 5A,B). Furthermore, the expression levels of genes related to reactive oxygen species (ROS) indicators, including VdSOD1, VdCAT1, and VdGSS, were assessed using real-time fluorescence quantitative PCR (Figure 5C). The analysis revealed a down-regulation in the expression of these genes in the ΔVdSti1 mutant in comparison to the wild type and complementation mutants. These results strongly indicate that VdSti1 is involved in *V. dahliae*’s response to H2O2-induced oxidative stress. The heightened sensitivity to H2O2 and the reduced expression of ROS indicator genes in the ΔVdSti1 mutant highlight the importance of VdSti1 in regulating oxidative stress responses in this fungus.

### 3.6 Deletion of *VdSti1* in *V. Dahliae* enhances the formation of melanin and microsclerotia

**Figure 6:**
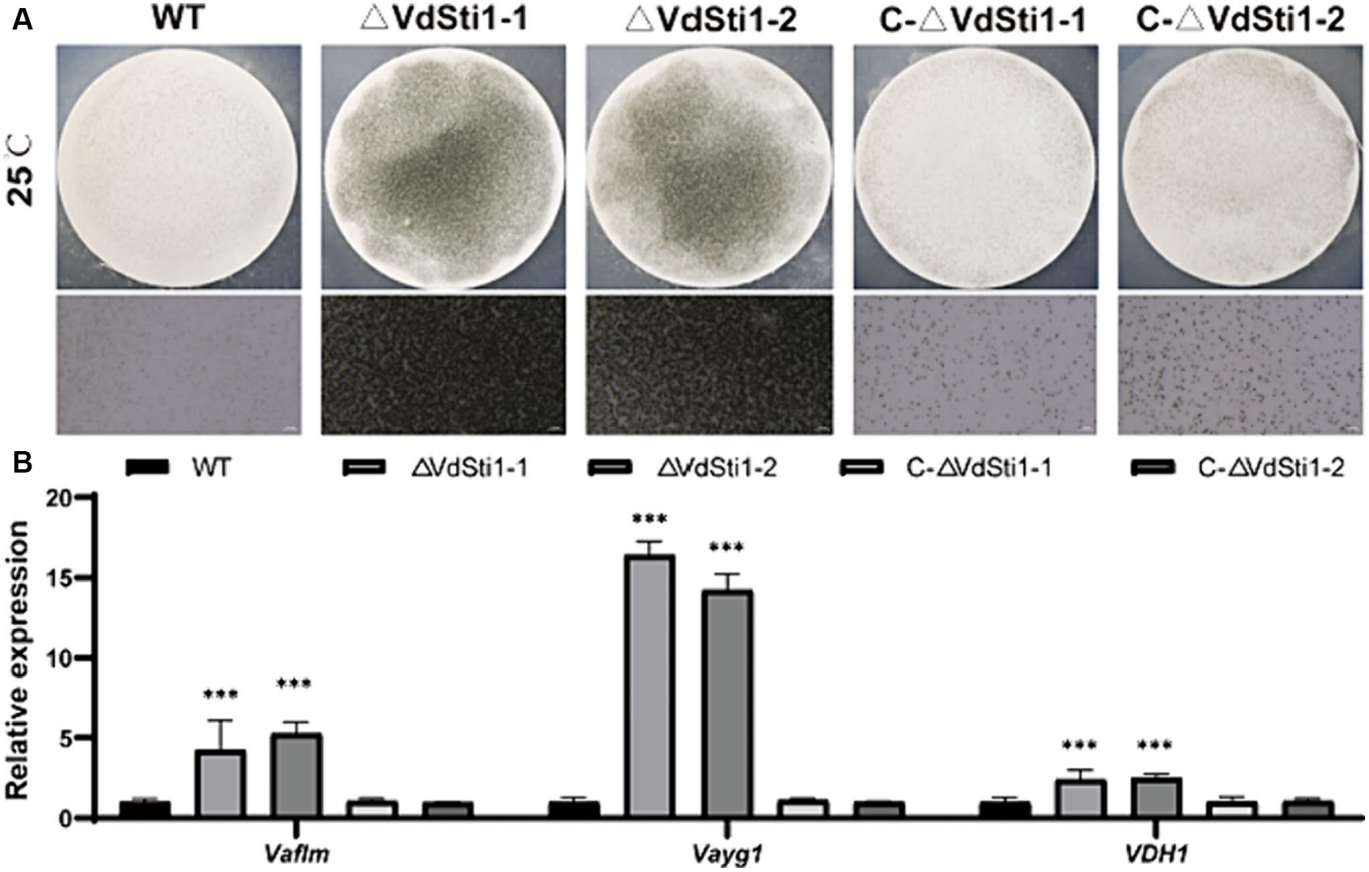
VdSti1 promoted the production of microsclerotia. **(A)** Growth of microsclerotia of wild type, ΔVdSti1 and C-ΔVdSti1 strains cultured on BM solid medium containing nitrocellulose membrane for 15 days. **(B)** Analysis using quantitative RT-qPCR to detect the expression of melanin-related genes VaflM, Vayg1, VDH1. Values represent means ± standard deviation of three replicates. The asterisks represent statistical differences performed by a *t*-test in comparison with the wild type strains (ns, *p* < 0.01,*p < 0.05, **p < 0.01, ***p < 0.001).

The ΔVdSti1 mutant exhibited elevated melanin production under both low and high temperature conditions. To explore the connection between VdSti1, melanin, and microsclerotia formation in *V. dahliae*, we assessed melanin and microsclerotia production after 15 days of incubation on solid basal medium (BM) with cellulose membranes. Results showed that the ΔVdSti1 mutant significantly increased melanin and micronuclei production compared to the wild type and complementation mutant strains (Figure 6A). Additionally, gene expression analysis of *VaflM* (VDAG_00183), *Vayg1* (VDAG_04954), and *VDH1* (VDAG_02273) associated with melanin and microsclerotia formation revealed significantly higher levels in the ΔVdSti1 mutant than in the wild type and complementation mutants (Figure 6B). These results suggest that VdSti1, as an Hsp90 co-chaperone, negatively regulates microsclerotia formation. The increased melanin production and upregulation of genes linked to melanin and microsclerotia formation in the ΔVdSti1 mutant imply the involvement of VdSti1 in modulating these processes in *V. dahliae*.

### 3.7 *VdSti1* positively regulates the pathogenicity of *V. Dahliae*

**Figure 7:**
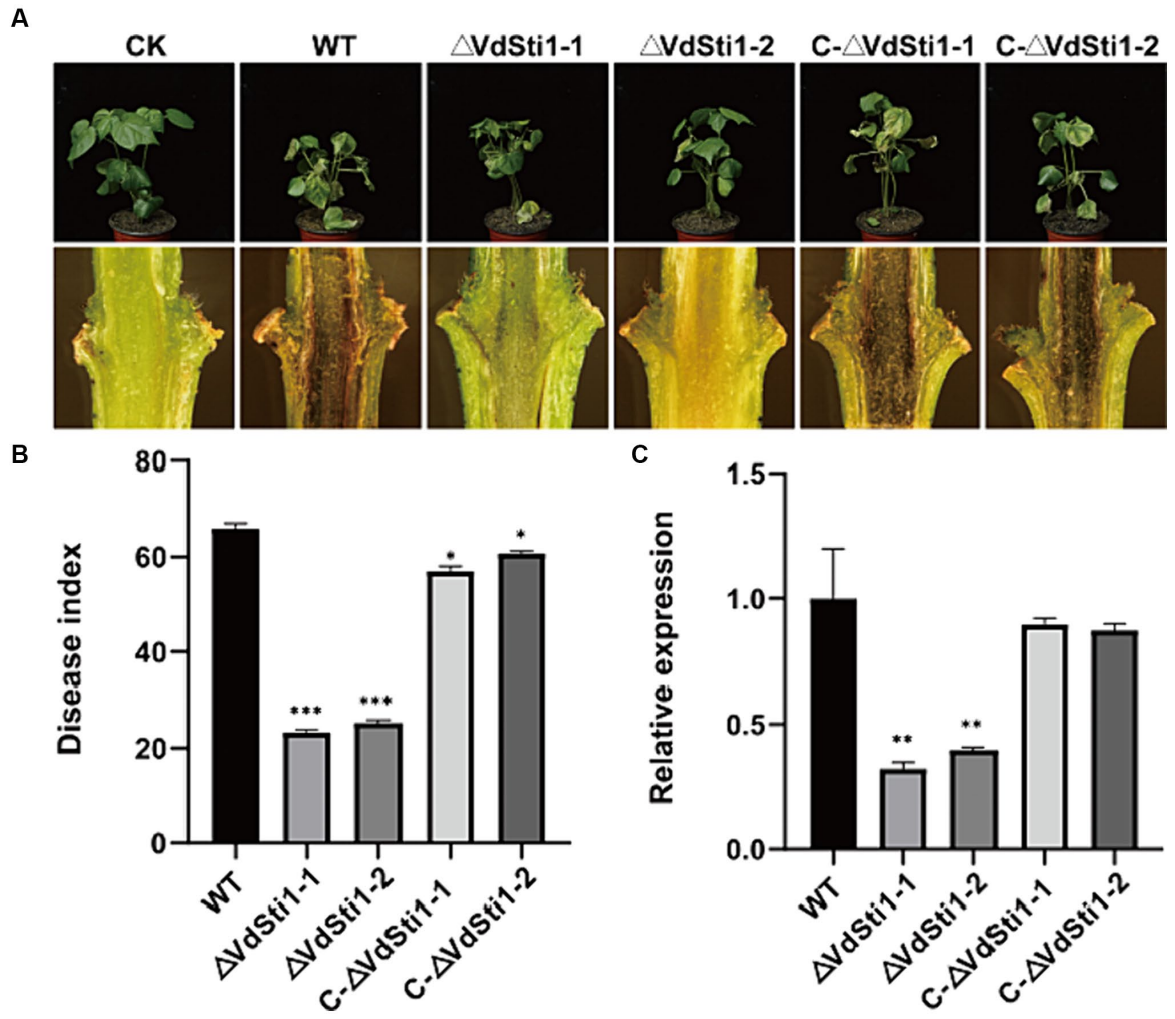
VdSti1 positively regulated the pathogenicity of *V. dahliae* in cotton. **(A)** Disease symptoms of cotton after the wild type, ΔVdSti1 and C-ΔVdSti1 strains infection. Vascular discoloration of the cotton stem tissue. Photographs were taken 21 days after fungal inoculation. **(B)** Disease index of cotton plants at 21 days after the wild type, ΔVdSti1 and C-ΔVdVdSti1 strains infection. **(C)** Fungal biomass in stems of cotton after the wild type, ΔVdSti1 and C-ΔVdSti1 strains infection at 21 days. *Vdβt* was used as the detection gene, and *GhUBQ7* of upland cotton was used as the endogenous control gene. Values represent means ± standard deviation of three replicates. The asterisks represent statistical differences performed by a *t*-test in comparison with the wild type strains (ns, *p* < 0.01,*p < 0.05, **p < 0.01, ***p < 0.001).

To further investigate the impact of the Hsp90 co-chaperone VdSti1 on the pathogenicity of *V. dahliae*, inoculation experiments were conducted using conidial suspensions of the wild type, ΔVdSti1 mutant, and C-ΔVdSti1 mutant strains, with pure water as a control. After a 21 days incubation period, clear symptoms of leaf yellow wilt and necrosis were observed in the wild-type and C-ΔVdSti1 mutant strains (Figure 7A). In contrast, the ΔVdSti1-1 and ΔVdSti1-2 mutant strains exhibited significantly reduced yellow wilt symptoms, leading to a substantial decrease in the disease index by approximately 0.35 and 0.38 times, respectively (Figure 7B). Examination of cotton stalks revealed less browning in those inoculated with the VdSti1 knockout mutants compared to the wild-type strain (Figure 7A). Furthermore, qRT-PCR analysis showed no significant difference in root fungal biomass between the C-ΔVdSti1-1 and C-ΔVdSti1-2 strains and the wild-type strain. However, both the ΔVdSti1-1 and ΔVdSti1-2 strains displayed a notable decrease in fungal biomass by approximately 0.32 and 0.40 times, respectively (Figure 7C). These results suggest that VdSti1 plays a critical role in positively regulating the pathogenicity of *V. dahliae*.

### 3.8 RNA-Seq analysis of *VdSti1* knockout mutants

**Figure 8:**
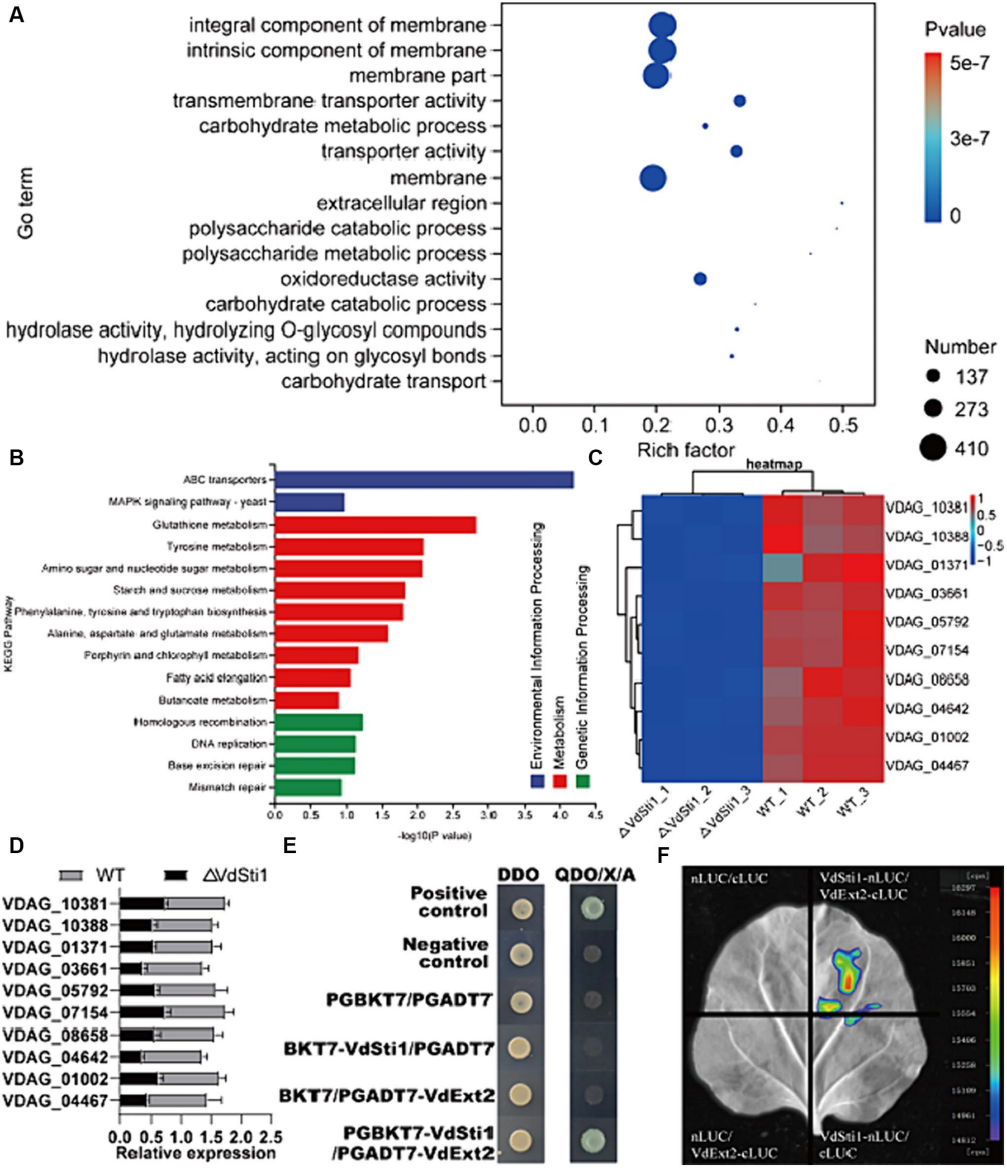
VdSti1 participates in multiple related pathways. **(A)** Gene ontology enrichment of DEGs from ΔVdSti1 versus WT. Gene ratio is the number of DEGs divided by the total number of genes associated with a specific pathway. **(B)** KEGG enrichment of down-regulated DEGs from ΔVdSti1 versus WT. **(C)** Heatmap of partially downregulated genes in knockout mutants. **(D)** Analysis using quantitative RT-qPCR to detect the expression of related genes. Values represent means ± standard deviation of three replicates. **(E)** Yeast two-hybrid assays of the interactions of VdSti1 with VdExt2. **(F)** The interaction of VdSti1 with VdExt2 was detected by LCI assay.

To gain a better understanding of the potential function of VdSti1 in *V. dahliae* strains, we analysed differentially expressed genes (DEGs) in ΔVdSti1 strains using RNA sequencing (RNA-seq). The RNA-seq results identified 2,627 DEGs in the ΔVdSti1 strain, including 1792 up-regulated and 835 down-regulated genes (Supplementary Figure S5). This result clarifies the gene expression changes resulting from the knockdown of VdSti1 in *V. dahliae*. To enhance comprehension of the down-regulated genes’ function, this study conducted Go term and KEGG enrichment analyses. The figure displays the top 15 significantly enriched Go term pathways, including integral component of membrane, intrinsic component of membrane, membrane, transmembrane transporter activity, transporter activity, oxidoreductase activity, carbohydrate metabolic process, and extracellular region pathways (Figure 8A). The pathways consist of differentially expressed genes (DEGs), with 186, 186, 194, 42, 42, 82, 30, and 8 genes, respectively. The figure shows the top 15 significantly enriched KEGG pathways, such as ABC transporters, MAPK signaling pathway-yeast, Glutathione metabolism, and Homologous recombination. It contains DEGs of 7, 6, 6, and 3, respectively (Figure 8B). We randomly selected ten down-regulated candidate genes in the enrichment pathway and analysed the expression levels of the wild-type and ΔVdSti1 strains. The ΔVdSti1 strain caused down-regulation of all 10 candidate genes in comparison to the wild-type and C-ΔVdSti1 strains (Figure 8C). The RT-qPCR results showed a correlation between the expression levels of the 10 genes and the RNA-Seq results (Figure 8D). These findings suggest that the knockout of VdSti1 in *V. dahliae* resulted in changes in the expression levels of other genes involved in different pathways with different biological functions.

To gain further insight into the role of VdSti1 in the pathogen, we cloned the CDS sequence of VdSti1 into the pGBKT7 vector as a bait. From a cDNA library of *V. dahliae*, which was subjected to cotton root baiting, we identified five proteins (VDAG_01750, VDAG_01827, VDAG_03209, VDAG_04454, and VDAG_10388) that may interact with VdSti1 (Supplementary Figure S6). Through yeast two-hybrid experiments, we confirmed that the exostosin-2 protein (VDAG_10388, named VdExt2) interacts with VdSti1 (Figure 8E). Furthermore, we confirmed the interaction between VdSti1 and VdExt2 by conducting LCI assays in N. benthamiana (Figure 8F).

## 4 Discussion

The conserved and multifunctional nature of the Sti1 protein in fungi is widely recognized. The Sti1 gene in *Verticillium dahliae* is highly conserved with Sti1 genes in other fungi, suggesting that its functions may be shared and conserved among different fungal species. Previous studies on Sti1 in other fungi have elucidated its important roles in multiple cellular processes.

VdSti1 knockout led to reduced ergosterol biosynthesis in *V. dahliae* and increased sensitivity to SDS and CR stress. The Sti1 protein acts as a co-chaperone of Hsp90, improving the association between Hsp70 and Hsp90 (Reidy et al., 2018). Although Hsp90 and its co-chaperones do not directly activate gene transcription, Sti1 proteins may be involved in relevant signaling pathways through interactions with Hsp90 client proteins (Gu et al., 2016). In *Neurospora crassa* and *Fusarium verticillioides*, the transcription factors CCG-8, ADS-4, and CSP-1 regulate the transcriptional responses of genes involved in ergosterol biosynthesis, a critical fungal-specific process (Sun et al., 2014; Wang et al., 2015; Chen et al., 2016). In *N. crassa*, the knockout of Sti1 led to the accumulation of toxic sterols and cell membrane stress following ketoconazole treatment (Gu et al., 2016). Sti1 positively regulated the stress response genes erg11 and erg6, which are involved in ergosterol biosynthesis, in response to ketoconazole. Similarly, the knockout of VdSti1 in *V. dahliae* resulted in azole sensitivity, indicating its involvement in ergosterol biosynthesis. Erg6 is essential for normal cell membrane function and is important for ergosterol biosynthesis (Gaber et al., 1989). Knockout of *VdUGP* and *VdEPG1* in *V. dahliae*, which affect membrane integrity, hindered growth under SDS stress (Deng et al., 2020; Liu et al., 2023). In addition, reduced ergosterol biosynthesis was observed in *V. dahliae* upon the knockout of *VdERG2*, emphasizing its role in ergosterol production (Lv et al., 2023). Our study demonstrated that the knockout of VdSti1 led to reduced ergosterol biosynthesis in *V. dahliae* and increased sensitivity to SDS and CR stress. These findings highlight the significant role of *VdSti1* in ergosterol biosynthesis and the maintenance of cell membrane and cell wall integrity in *V. dahliae*.

VdSti1 negatively regulates the formation of melanin and microsclerotia. *V. dahliae*, as a fungal pathogen, is capable of surviving in the soil as microsclerotia for extended periods, even in the absence of a host (Bui et al., 2019). These microsclerotia can function as a reservoir and germinate in the presence of suitable invasive plant roots, giving rise to conidia that colonize the plant. Therefore, microsclerotia play a critical role in the invasion process of *V. dahliae*. In this regard, *VdPbs2*, *VdHog1*, and *VdMsb2* have been identified as important regulators of microsclerotia formation through the HOG pathway in *V. dahliae* (Bahn, 2008; Tian et al., 2016; Wang et al., 2016b; Yu et al., 2019). In our study, we found that VdSti1 negatively regulates the formation of melanin and microsclerotia in *V. dahliae*. However, it remains unclear whether VdSti1 mediates this regulation through Hsp90 client proteins. Future research should focus on investigating the specific mechanisms by which VdSti1 influences melanin and microsclerotia formation, including its potential interactions with Hsp90 client proteins.

The VdSti1 knockout mutants in *V. dahliae* exhibited increased sensitivity to hydrogen peroxide. When plants are infested with pathogens, they produce hydrogen peroxide (H2O2), which causes oxidative stress for the invading pathogens (Fassler and West, 2011). Knockout of Sti1 in *N. crassa* negatively regulates H_2_O_2_ resistance (Gu et al., 2016). In *V. dahliae*, the deletion of *VdSkn7*, a gene involved in oxidative stress response, results in increased sensitivity to H_2_O_2_ (Tang et al., 2017). In our study, we observed that the *VdSti1* knockout mutants in *V. dahliae* exhibited increased sensitivity to hydrogen peroxide. These findings suggest that *VdSti1* likely plays a role in the oxidative stress response of *V. dahliae*.

We conducted a comprehensive investigation of the function and pathogenicity regulation of *VdSti1* in *V. dahliae*. VdSti1 is a positive regulator of the pathogenicity of *Verticillium dahliae*. This suggests that VdSti1 may inhibit the defence response of host plants during *V. dahliae* infestation by modulating Hsp90 and related signalling pathways. RNA-seq analyses were performed on ΔVdSti1 and wild-type strains to investigate its mechanism of action in *V. dahliae*. A total of 835 genes were identified as down-regulated, with 82 of them enriched in the oxidoreductase activity pathway. Extensive research has focused on the dual nature of ROS in biological systems. On one hand, it has the potential to cause cellular damage, while on the other hand, it serves as a signalling component for important developmental processes (Lambeth, 2004; Heller and Tudzynski, 2011; Lambeth and Neish, 2014; Sies and Jones, 2020). NoxA, the NADPH oxidase of ROS, plays a crucial role in virulence by mediating responses to different types of cellular stress (Vangalis et al., 2021). In addition, six down-regulated genes were enriched in the MAPK signalling pathway. The plant MAPK plays a crucial role in the signalling pathway of plant defence against pathogens (Zhang and Klessig, 2001; Meng and Zhang, 2013). The current study revealed that VdSti1 may further promote *V. dahliae* plant infection by altering *V. dahliae* resistance-related pathways through the regulation of Hsp90.

It is hypothesized that VdSti1 in *V. dahliae* regulates pathogenicity by modulating Hsp90 function and related signaling pathways. This includes regulating micronucleus formation, ergosterol biosynthesis, cell membrane and cell wall integrity, and participating in oxidative stress responses. A comprehensive investigation of the function and pathogenicity regulation of VdSti1 in *V. dahliae* was conducted in this study. The findings offer valuable insights and a basis for additional research into the pathogenesis and control of fungal diseases. A thorough comprehension of VdSti1 will be essential for supporting sustainable crop production and efficient crop disease management.

## 5 Conclusion

Our study offers valuable insights into the function and mechanisms of *VdSti1* in *V. dahliae*. By clarifying its role in pathogenicity and stress responses, we contribute to a comprehensive understanding of this important fungal pathogen. These findings expand our knowledge of Sti1/Hop family proteins in fungal pathogens and open new avenues for precision disease management and crop production. Targeted research and development of new drugs can be carried out in the future based on the role of VdSti1 in ergosterol synthesis. Future research should concentrate on investigating the underlying molecular mechanisms and potential targets for intervention based on the role of *VdSti1* in *V. dahliae* pathogenicity.

## Data availability statement

The datasets presented in this study can be found in online repositories. The names of the repository/repositories and accession number(s) can be found in the article/Supplementary material.

## Author contributions

YW: Writing – original draft, Writing – review & editing. JZ: Writing – review & editing, Writing – original draft. FW: Data curation, Writing – review & editing. YZ: Investigation, Writing – review & editing. LZ: Funding acquisition, Writing – review & editing. ZF: Conceptualization, Investigation, Supervision, Writing – review & editing. HF: Conceptualization, Data curation, Formal analysis, Funding acquisition, Investigation, Methodology, Project administration, Resources, Software, Supervision, Validation, Visualization, Writing – review & editing.

## Footnotes

^1^ https://www.ncbi.nlm.nih.gov/tools/primer-blast/

^2^ https://www.ncbi.nlm.nih.gov/

^3^ https://www.ncbi.nlm.nih.gov/genome/832
