# Supplementary material for: The role of VdSti1 in Verticillium dahliae: insights into pathogenicity and stress responses
Source: Front Microbiol. 2024 Apr 4;15:1377713. doi: 10.3389/fmicb.2024.1377713 (PMC11024458; doi:10.3389/fmicb.2024.1377713)
Supplement: Supplementary file 1 [file Data_Sheet_1.docx]

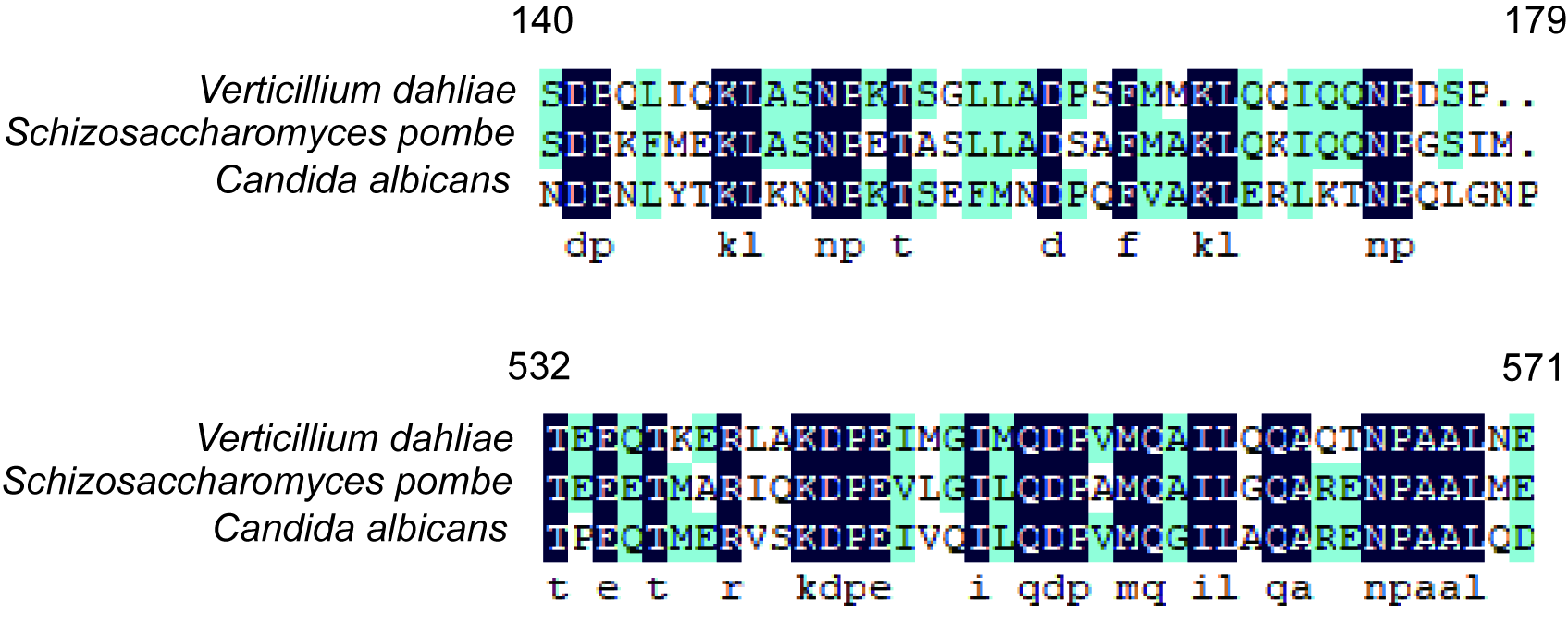


**Figure S1** The amino acid sequence alignment of the Sti structural domain(genotype of Sti1 in *Verticillium dahliae*、*Schizosaccharomyces pomb*e and *Candida albicans*).Numbers represent the position of the amino acid (aa) residues, and sequence alignments were performed with DNAMAN.


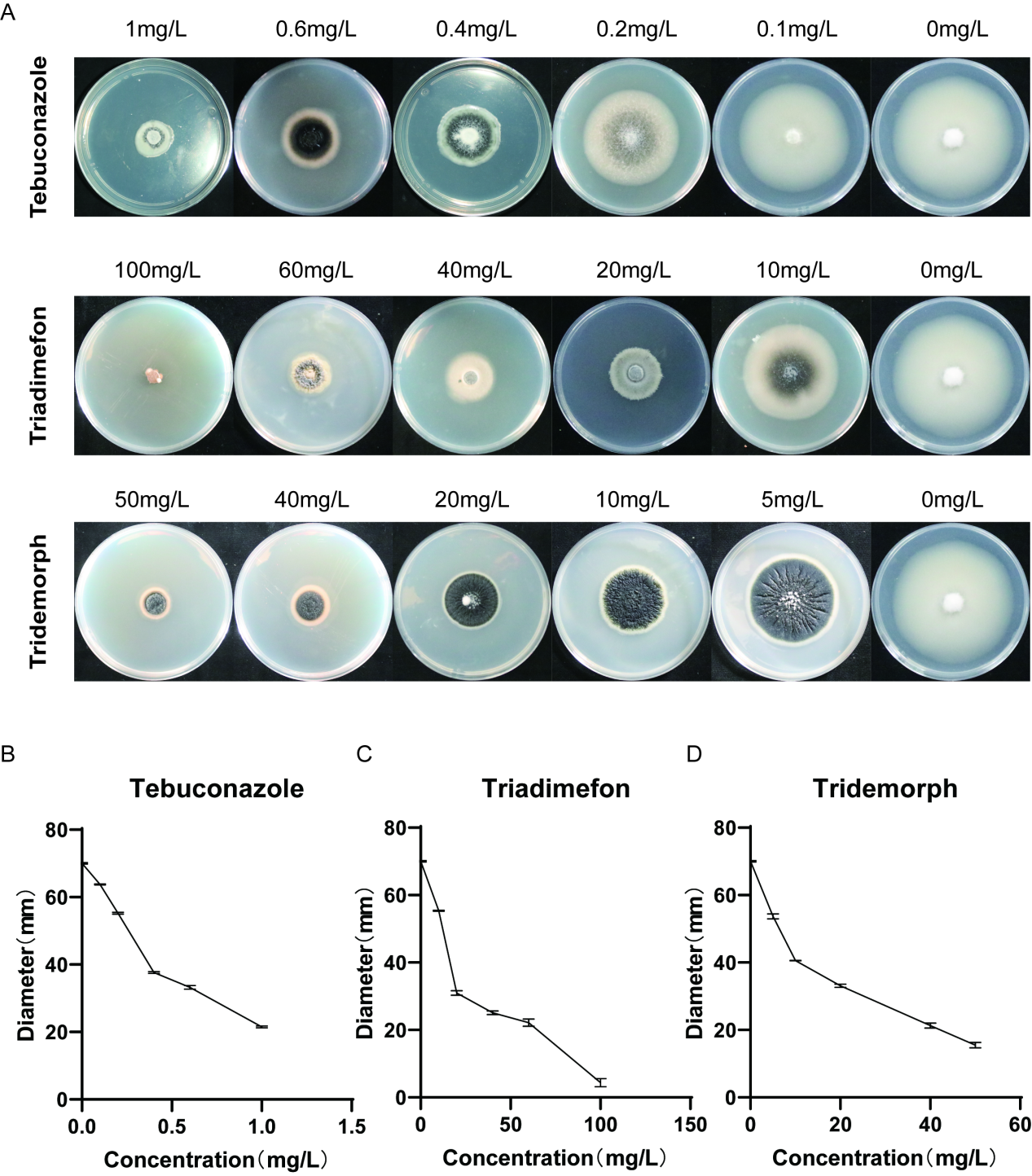


**Figure S2** The growth of Vd080 mycelium under different drug concentrations.(A)Vd080 strains were cultured on PDA plates supplemented with tebuconazole , triadimefon and tridemorph at 25 °C for 14 days. Scale = 1 cm.(B、C、D)Colony diameter of all strains.


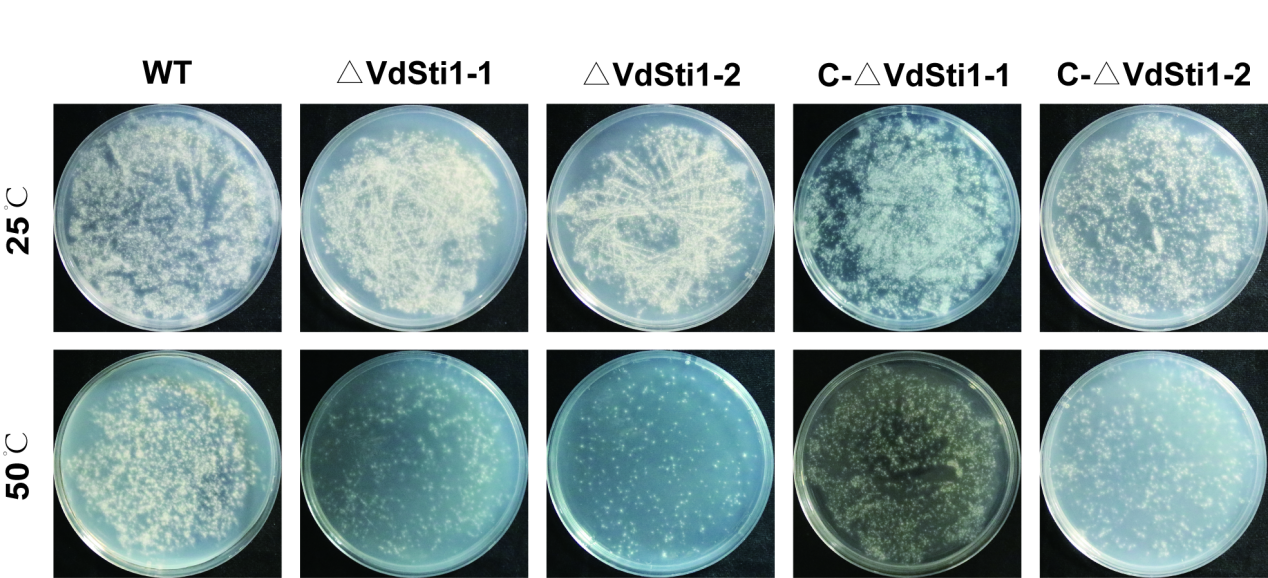


**Figure S3** Number of mutant spore germination before and after 50°C heat shock treatmentin in the wild type, ΔVdSti1 and C-ΔVdSti1 strains.


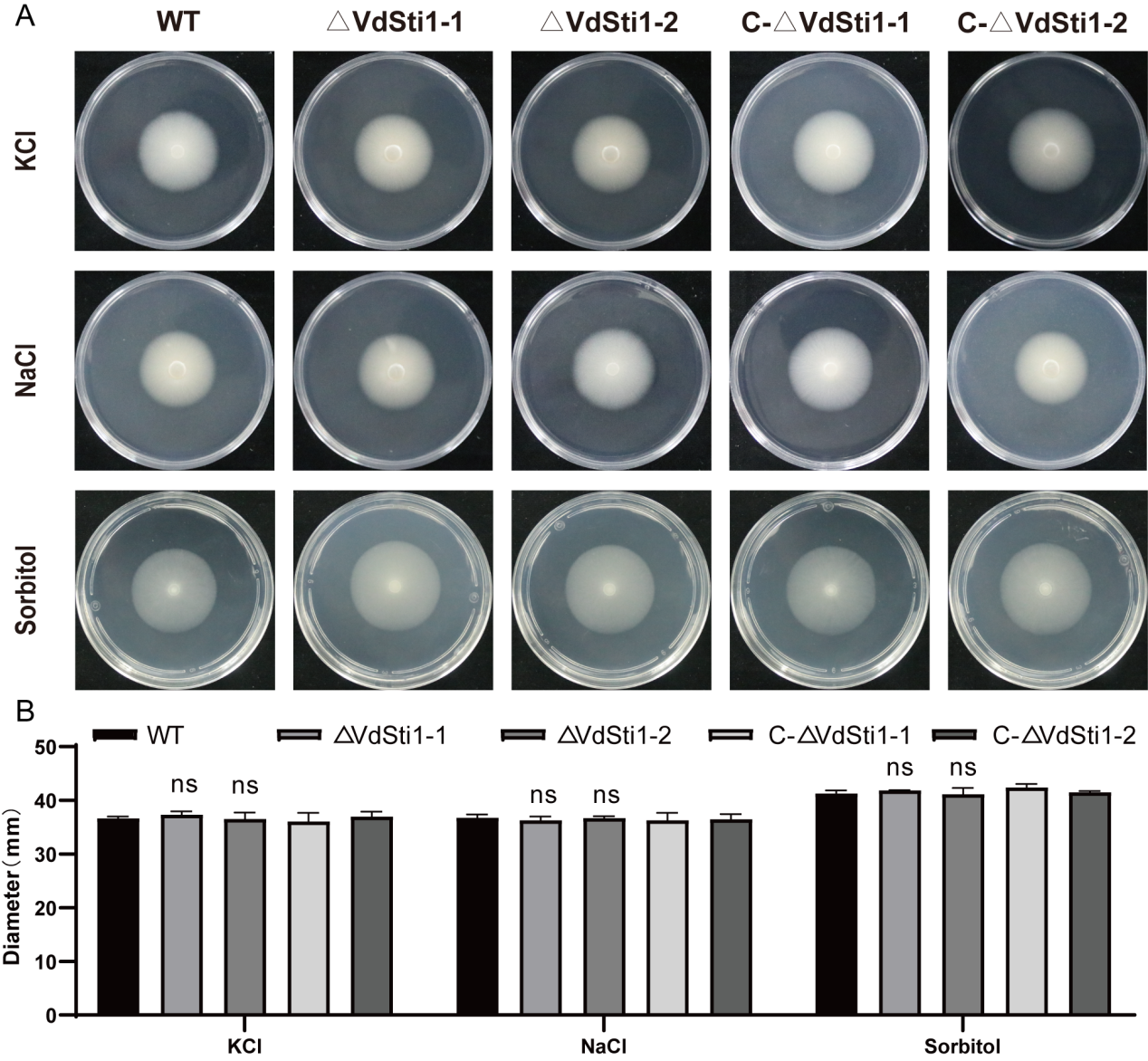


**Figure S4** The growth of the wild type, ΔVdSti1 and C-ΔVdSti1 strains under osmotic stress（A）All strains were cultured on PDA plates supplemented 1 M/L KCl, 1 M/L NaCl, 1 M/L sorbitol at 25 °C for 14 days. Scale = 1 cm.(B、C、D)Colony diameter of all strains. Values represent means ± standard deviation of three replicates. The asterisks represent statistical differences performed by a t-test in comparison with the wild type strains. (ns,p < 0.01,*, p < 0.05, * *, p < 0.01, * * *, p < 0.001)

**
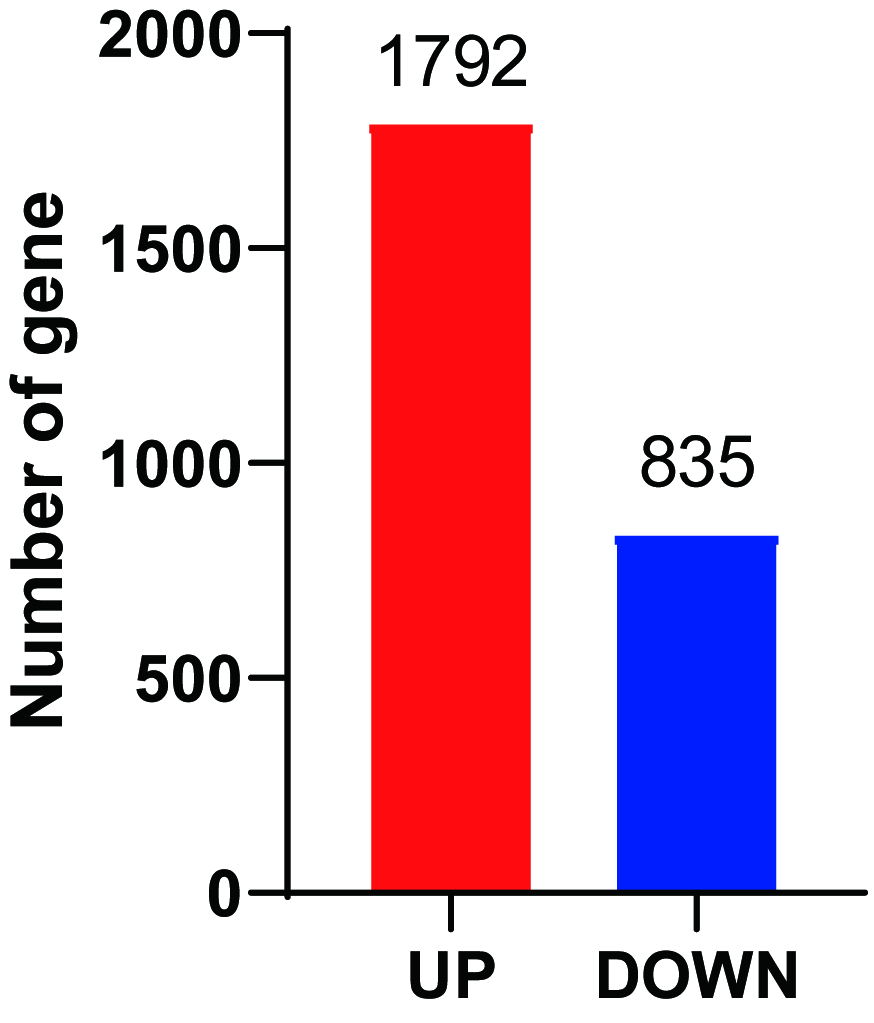
**

**Figure S5** The RNA-seq results of differentially expressed genes (DEGs) in ΔVdSti1 strains.

| Accession No. | Descripition | Sequencing Reads |
| --- | --- | --- |
| VDAG_01750 | cysteine proteinase | GATGTTCATTCCTGTCATCAGGTCCATGCCATTCGAGAGCGTGAGTTTGGACTGAAGACTGACCTGGATTGCTATCAATTTACTACCTGCCCGAATCATCAAGACAAACCCTACGATGGGAGCCCAACTCTCAACGCATGACGCTGCCGATAAGGCCCTTGTCGATCGCCTCGAAGGTCTTCAGGTCCGCAGCACGTCCAACGACTTTGTCCATGTATCTAAGGATGGGTCGCAGAGCGAGAAGCAGGCTCTTTCCAGACTTATCCATCGCGAACCAGAGGGTATCTCTGTCAAGCTTGTAGAAGACTGGCAGAGCACCTTTCTCAAGGACCCCAAGAACAGGTTGGCCCTTTCTGCCCTCAGCAATGCCGATCCGCGTACCGTCCTGACGTCTACACCCGCCACGCTGTCTGACCCTCAAATATTCAACGTCCGCATCCCCCTCGAGGGAACGCCGATAACAAACCAGCGCTCTTCCGGCCGCTGTTGGCTCTTCGCCTCGACGAATGTTTTCCGTGTTGCCCTCGCCCGCCGCTACAACCTTGCGGGCTTCGAACTATCACAGTCCTACCTTTTTTTCTGGGACAAGCTCGAGAAGGCCAACTGGTTCCTCGAGCAGGCCATTGCCACGGCTGCCGATCATGACCTCGACTCCCGACTCGTGCAGCACCTCCTCGGTGACCTCATTTCAGATGGCGGGCAATGGGACATGGTCTACAACCTCGTCGACAAGTATGGTCTCGTGCCTCAATCCTTGTACCCGGACTCGTGGAATGCCCAGAATTCGGGTGTCCTCAACTCCGTGCTCAAGACGAAGCTGCGCGAGTATGCCCTCACCCTCCGCTCTCTTGTCAACGAGCAGAGCGCTACTGCTCAAAGCGTCAAGGACGCCAAGTCCAAGATGCTCCAGTCCATCCTGGGAATCTTGACCCTGACACTTGGCGCACCGCCACACCCGCACGAGGCCTTCGTTTGGACCTACGAAGACAAGGACGGCAAAGCCCATGAGCTGACATCGACCCCGCGCGCCTTTGCCCGCGACATCTCCTCGAGTCAATGCCGCGTTTCGAGCGCCACCATTGCAGGTATGGTCTCGCTCGTGCACGACCCGCGTCACGACCCCTTGACCCTCTTGACCGTCGACCGCCTCGGCAACATTGTGGGCGGCCGCCCAGTCACCTACGTCAACGTCTCCATTTCCGCCCTCAAGGCAGCCTGCGTGCGCCAGCTTCGCGCCGGCCTCCCCATCTTCTTCGGCTCTGATGTGGGCAAATTCAGCTCCTCGCGCTCCGGTGTCATGGACCTCGACCTTGTCGACTACGAAGTCGGCTTCGATGTGAGCCTGCTCGCAATGTCCAAGGCCGATCGCCTGCGCACGGGCGAGTCGCAGATGACCCACGCCATGGTCTTGACGGCCGTCCACATCGACGACAAGACGGGCCGCAGCGTCCGCTGGCGCGTCCAAAACAGCTGGGGCGAGGCCGCCGGCGACAGGGCTGGTTCGTCATGAGCGACGCCTGGATGGACGAGTTTGTCTACCAGGCCGTCATCGATCCCCGCTTCCTCGACAAGGAAACAAAGGACGTCCTCAGGCAGGAGCCGGTCGTGTTGCCGCTGTGGGATCCCATGGGCAGCCTGGCATGAGGGGCAACCTATTTCAGGAGCAGGTTTGCAATCTGGTCTGATAAGTTATAGACAGTATGGCTCCCGTCGTTGAGTCGCATTACTACACCAGGCCCGATCATACCTACTCATTGGAGAATTGAACACGCACAGCGGTTTGTCTGTCGTGCGCTACTTGTGAAGACCTGCCCCGGTGATCTGATACAGTAAAGGACCGACCATGTCTACATGTTCAAGCAGTGCAATACGCAGGCCTTGAGTCGAGGTTCCCTGCTTGGATCCGAACACAATGCAGTTCGTGCAAGATTTCGTTGAGTGGACTGGCAACTTGGTCAACCAAAGTTGAGTACCATACCAAGACCATGCCCTAGAGCCGATTTGTTACAAGCACCACTGTTCGCTAGTAGTATTTGCTCGTCGTGGCAGCACCCAGC |
| VDAG_01827 | [tubulin gamma chain](https://www.ncbi.nlm.nih.gov/gene/20703290) | GGAATGAGATTATCAGTGATCCATCTTCGCTCTCCTGAATCAGCGATGCAGTCAGGGACTGAGAATGTCCCTCCCTGGGTAGTAGAACGACTGGTAGCGATTGGTGCTCCGAAGGCCCAATCAGGAAGCCAGGGGTGAAGGTAGGTACCCAACACTTATCGATAAGGGAGGAACGCGTCGAGCATCGCGGGTCCGCATCAATTGTGCATGACAGCAGGTGACGACGGCTCTACTACCAACGCGCTCTCGACAAGCCATACGCCAGCTATCAGCATGCCGAGAGAGATCATCACGATTCAGGCCGGCCAGTGCGGCAACAGCATTGGCAGTCAATTCTGGCAGCAATTATGTCAGGAACATGGCATCAGTCAAGATGGAAACCTTGAAGACTTCGCGACCGAGGGTGGCGATCGAAAAGACGTCTTTTACTATCAGAGCGACGACACGAGATACATTCCAAGAGCCATTCTGATCGATCTTGAACCACGCGTCATCAACGGCATACAGACAGGACCGTACAAGAACATATACAATCCCGAAAACTTCTACGTCAGCAAAGATGGTGTGGGTGCAGCCAACAACTGGGGTGACGGCTACCAGACTGGCGAGAAGGTGTTCGAGGACATCATGGAGATGATTGACCGTGAGGCCGATGGTAGCGATTCGCTCGAAGGCTTCATGATGCTGCACTCCATCGCAGGTGGCACTGGATCTGGACTTGGCTCGTTTCTCCTCGAGCGTCTGAATGACCGATTCCCGAAGAAGATCATACAGACGTATTCCGTCTTCCCCGACACAACGAACGCAGGCGATGTCGTTGTTCACCCCTACAACAGCGTGCTGGCCATGCGACGTTTAACACAGAATGCGGACTCAGTGGTGGTGCTCGACAACGGTGCTTTGTCAAGGATCGCCGCCGACAGACTGCACGTCCACGAGCCTTCCTTTGCGCAAACTAATCAACTTGTTTCCACTGTCATGTCTGCGAGCACCACGACCTTGAGATACCCGGGATATATGCACAACGACCTTGTCAGCATCCTAGCCTCCCTCATCCCGACCCCACGCTGTCACTTCCTGATGACATCCTACACACCGTTTACTGGCGACCAAGTCGAGCAGGCAAAGACTGTTCGGAAGACGACAGTGCTGGACGTGATGAGGCGGCTTCTTCAACCTAAGAACAGGATGGTGTCGACGCAACCTGGCAAAAAGAGCTGTTACATCTCTATTCTCAACGTCATTCAAGGCGAAGTCGACCCGACTGATGTCCACAAGAGTCTTCTTCGCATCCGAGAGCGAAGATTGGCAACCTTTATCCCCTGGGGTCCTGCAAGCATTCAGGTCGCATTAACAAAACGAAGCCCTTACATTCCAATGGCACACCGCGTTAGCGGGTTGATGCTAGCTAACCACACGAGCATTGCCACGCTGTTCAAACGAATTGTTAAGCAGTTTGATGGCATGCGGAAACGAAATGCCTTCATGGAGGGCTACAAGAAAACGGCTCCCTTCGCAGAGAACCTCAACGAATTCGACGAGTCTCGTCAGGTGGTGCAGGACCTGATCCAGGAATATGAGGCGGCCGAGGACGCTAATTACCTGAACCCTGAAGCAGAAGTTCCAACTTCCGCAGAGACAGACAAGAGATTGGGCTGAGGATCCCGGGAAGAAAGCGTCGCGTAACTGAGTGGCAATGGCGCGCCAAGAATGAAGGCATGACATGATTGTACCTGAGCGCTTCGATATGACCAGGTTTCTGGCATCTTCCATTGGGAGAGTTAGGCGTTTGGGGCTAGAGCATCCGTACCTAGGATCGCGACGTGAGGGCAATAGCTTGAAACTATCTATTTGACAAACCGTGCGCGGC |
| VDAG_03209 | [alternative oxidase](https://www.ncbi.nlm.nih.gov/gene/20704672) | ATGCTCTCAGCAAGAACATCCACCAAGCTCTGTGCCCCTCGGCAAGCGGCCCAACTCGCAAGAGTTGTAGCCTTGTCCAGCAGCGGCCATATCTCCATCCTCGGCCACCCAGCAACCCTGCGAACGACGACTCTCTACCGCCCGTCCTCCCAGCGCAACTTTTCGAGCACCCCAGCCTCCCGCCTCCGTGACTTCTTCCCAGCCAAGGAGACGGAACAGATCCGCAAGACGGCGCCGGCATGGCCGCACGAGGGTTACTCCGAGGCCGACATGCTCGCCGTCGTGCCGGGCCACCGCGTCCCCGAGACCTGGGGCGACTGGGCTGCGTGGAAGTTTGTGCGCGTCGCCAGGTGGACGATGGACCGCGCAACGGGCTTGAAGCCTGAGCAGCAGGTTGACAAGAAGAATCCCACCACCGCCGTGGTGGCCAATGAGCCCTTGACAGAGGCACAGTGGCTCGTACGCTTCATCTTCCTGGAGAGCATAGCAGGAGTCCCAGGCATGGTCGCTGGCATGCTCCGCCATCTCGGCAGCCTTCGTCGCATGAAGCGCGACAACGGCTGGATCGAGACGCTGCTCGAGGAGTCTTACAATGAGCGCATGCATCTGCTGACCTTTATGAAGATGTCGGAGCCGGGCTGGTTCATGAAGGTCATGCTCATCGGTGCTCAGGGCGTCTTCTTCAACGGCATGTTCTTGTCGTATCTCGTCTCGCCCAAAATCACGCATCGCTTCGTCGGCTACCTCGAGGAGGAGGCCGTGCACACGTACTCGCGCTGCATCCGCGAGATCGAGGAAGGCCAACTGCCCAAGTGGTCGGATCCCAACTTTAACATTCCCGACCTGGCAGTGCAGTATTGGAACATTCCGGAGGGGAAGAGAACGATGCGTGATCTCATACTATACATCAGAGCTGATGAGGCTGTTCACCGCGGCGTCAACCACACGTTGAGTAACCTCAACCAGAATGAGGACCCAAATCCCTTCACCAGCGAGTACAAAGACGGTCACAAGCCGGCAGCAGCTCTCAAGCCGGCTGGGTACGAGAGGGCTGAGGTCATCTAG |
| VDAG_04454 | [DNA damage checkpoint protein rad24](https://www.ncbi.nlm.nih.gov/gene/20705917) | ATGGGCCAAGAAGATGCCGTTTACCTGGCCAAGCTCGCCGAGCAGGCTGAGCGTTACGAGGAGATGGTCGAGAACATGAAGATTGTCGCCGGTGAGGACCGCGATCTGACTGTCGAGGAGCGCAACCTCCTCTCTGTCGCCTACAAGAACGTTATTGGCGCCCGCCGTGCCTCTTGGAGGATCGTTACTTCCATTGAGCAGAAGGAGGAGTCCAAGGGCAACTCATCCCAGGTTGGCCTCATCAAGGAGTACCGCCAGAAGATCGAGGCCGAGCTCGCCAAGATCTGTGAGGATATCCTTGAGGTCCTCGACAAGCACCTGATCCCCTCGGCCAAGTCTGGAGAGTCCAAGGTCTTCTACCACAAGATGAAGGGCGACTACCACCGTTACCTGGCCGAATTCGCCATTGGCGACCGCCGCAAGGACTCTGCTGACAAGTCGCTCGAGGCCTACAAGGCTGCCACTGAGGTCGCCCAGACCGAGCTTCCTCCTACCCACCCCATCCGTCTTGGTCTCGCCCTCAACTTCTCCGTCTTTTACTACGAGATCCTCAACGCCCCTGACCAGGCTTGCCACCTGGCCAAGCAGGCCTTTGACGATGCCATTGCTGAGCTCGACACCCTGAGCGAGGAGTCCTACAAGGACTCGACCCTGATCATGCAGCTGCTGCGTGATAACTTGACCCTGTGGACCTCGTCGGAGGCCGAGCCCGCCCAGACCGGATCCGGTGAGAGCGCCGCACCTAAGGAGGCCGCTCCCGAGGCGACCGAGGCACCGGCCGCGGCCGCGAGCACCGAGGAGGCCCCCAAGGCCGCCGAGTAA |
| VDAG_10388 | [exostosin-2](https://www.ncbi.nlm.nih.gov/gene/20711851) | GGCCAGGCTTTATTTGACTGACCTGCCGCCCCCTGCCCTGGCATCATCTCAAACCCACTTGCCCCGAGACACCAAGCCTGGTTCAAGCCGCAGGACAGGACGAAAGGGAACAGTGGCTGGATTCACTTTCAACTGTCGACTTGCCTTCTTTCAACCTTCTTCCCCGCGACATCGTTATCGACATCGACATCGCGACACGTTGTTGGAGCACCCTCTCCATCGCCGGGCACCTCGACCACACTCTCTTTCTCCCCACCCCCCGCCCGTCCAGAGAATCCCCGACATTTGATGCCCGCCATGTACAAGTCCGATGGCACCACGACGACCTCGGTCCTGAAGAACCTGCCCCAGTGGCTCACCAAGCGGCACGTCCTCTTCGCCCTCGCCCTCGCCACCAGTCTCCTTCTTCTATTTTCCTTCAACTCGTGGGGCCCTGAAGTGCACCTCCCCCACGTCACCACCGGTACCGCCGATGCCCAGAAGAGCGCTTCCTCCAACAGCGACAATGGCCATTGCCATTCAGACGTACCGGCGTCCCGATGAACTGAACCAGACGCTCCACCTCCTCACAGACAACGTGATTCCCTCTCTGCACGAAATCGTCATCGTGTGGAACGACCTCGAGTCCACGCCCCCGCCCAACTTTGTCTCCGCCCACGGCGTCGGCGTTCGTTACCGTGTCTCCCGCCGCAACTCGCTGAACGAGAAGCTGTTCCCCGACCCAGAGTACAAGACCAAGGCCATCCTTCTTTCCGACGACGATGTCCACTACCCGCCCGCCGACCTCGACTTTGTCTTCCAGACCTGGCGCAAGTACGGCCGCCACCGCCTCACTGGTGCCTTTGCCCGCTGCGTCGACACCCCTCGCGGGCCCGGAAGCTACCAGTACAGCCTTTGCCGCGAGAAGGGTCGCTCCGAATACGCCCTTGTGCTGACGGGCCTCGCTTTCGCTCACATTGAGGTGCTCGACTACTTCTCCTCCACCGACCCCCTCATGACCCGCCTGCGCACTGCTATTGATGAGCACTTCAACTGCGAGGATATCGCACTCAACTTTGTCTCCTCCATGCTGTCCTGCGAGGGCCCCCTCGAGGTTCACGGCATGGGCCTGCCCGTCAACACGGAGCCCAAGTCGGGCATTAGCCGCAAGCCGGGCCACGCCAAGCTGAGGAACGACTGCCTGCGCGACTTCTCCGACTGGTTCGGATACATGCCGCTGCAGAACACGACCGAGCGCATTGTGCAGGGTATCTTGGCGATATAA |


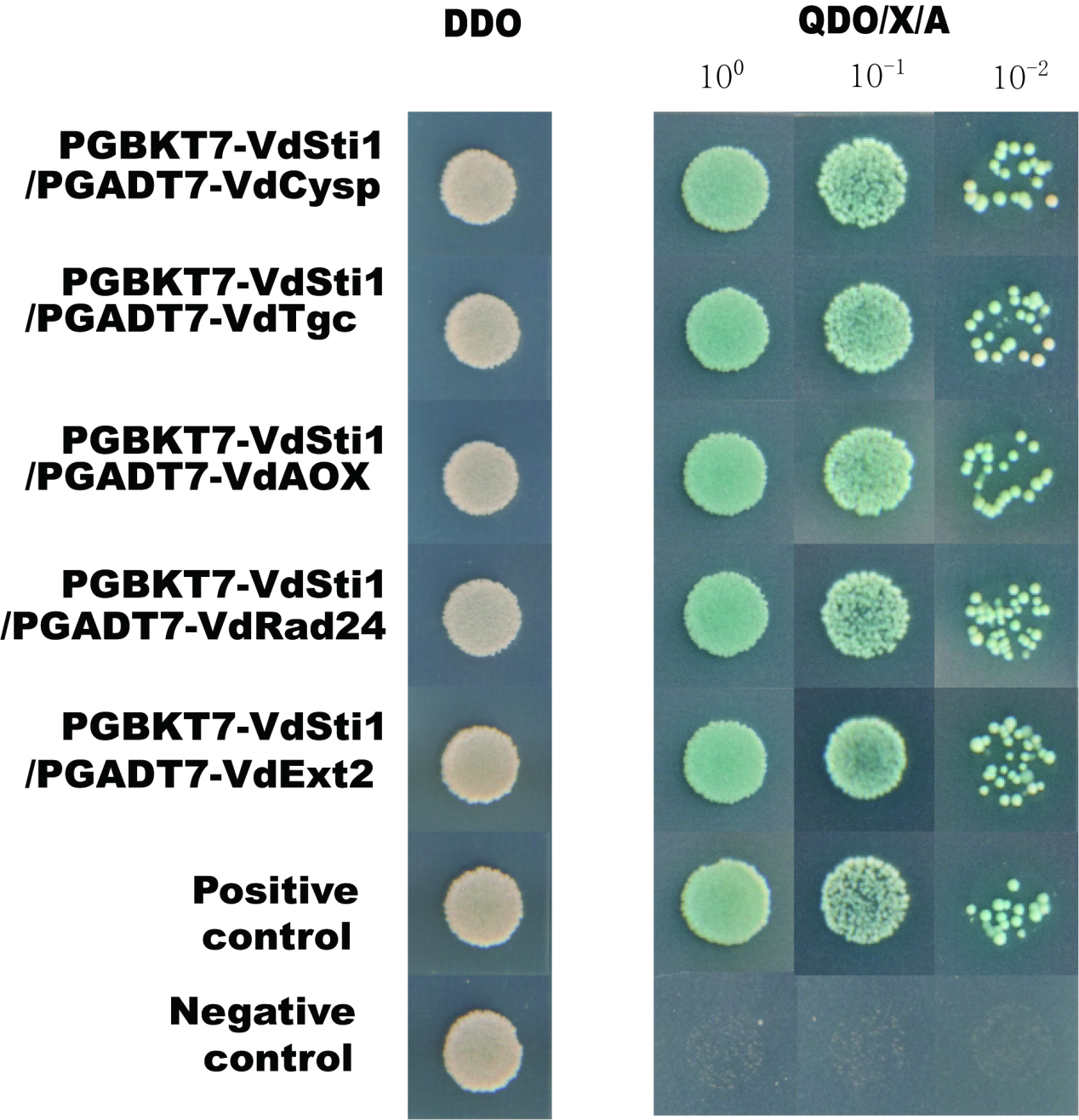


**Figure S6** Five proteins that may interact with VdSti1

**Table S1**

| Primers name | Primer sequence (5’-3’) | restriction site | use |
| --- | --- | --- | --- |
| VdSti1-F | ATGGCTACCGCAGACGAGCTCA |  | PCR |
| VdSti1-R | TTACCGACCAACGCGAATAAC |  | PCR |
| Hyg-F | GACGTTAACTGATATTGAAG |  | PCR |
| Hyg-R | CTATTCCTTTGCCCTCGGACG |  | PCR |
| B303-VdSti11-UP-F | GGACCGGACGGGGCGGTACC CAGGTGTACGAATAAAACGG | *Kpn*I | deletion of VdSti1 |
| B303-VdSti1-UP-R | CTTCAATATCAGTTAACGTC CAAGAGCCGCGTGTCCGTTG |  | deletion of VdSti1 |
| B303-VdSti1-Hyg-F | CAACGGACACGCGGCTCTTG GACGTTAACTGATATTGAAG |  | deletion of VdSti1 |
| B303-VdSti1-Hyg-R | ACTGCTTGTGTCCGGCTGAG CTATTCCTTTGCCCTCGGACG |  | deletion of VdSti1 |
| B303-VdSti1-DOWN-F | CGTCCGAGGGCAAAGGAATAG CTCAGCCGGACACAAGCAGT |  | deletion of VdSti1 |
| B303-VdSti1-DOWN-R | TTAATTAAGACCCGGGACTA GTTCGTCCAAGGAAGAAAGA | *Pac*I | deletion of VdSti1 |
| BIA1302-VdSti1-C-F | ACGGCCAGTGCCAAGCTT  AGAAGTTTCTGAACAGCGAT | *Bamh*I | complementation of VdSti1 |
| BIA1302-VdSti1-C-R | ATGGCCTCCTCCGAGAACGTCTCCGACCAACGCGAATAACG |  | complementation of VdSti1 |
| BIA1302-VdSti1-RFP-F | TTATTCGCGTTGGTCGGTAA GACGTTCTCGGAGGAGGCCAT |  | complementation of VdSti1 |
| BIA1302-VdSti1-RFP-R | ATTCACTAGTCAGGATCC  CAGGAACAGGTGGTGGCGGCC | *Hind*Ⅲ | complementation of VdSti1 |

**Table S2**

| Primers name | Primer sequence (5’-3’) | use |
| --- | --- | --- |
| GSS-qPCR-F | CGTCACAGCGGAGCAGTAC | qRT-PCR |
| GSS-qPCR-R | GCACATTGATGGCGAGCAT | qRT-PCR |
| SOD1-qPCR-F | CTTCACTTCAGCAGCAACCTC | qRT-PCR |
| SOD1-qPCR-R | CAGTAGGCACGGGAACGAG | qRT-PCR |
| CAT1-qPCR-F | TTCCAACAGCACCAGCAA | qRT-PCR |
| CAT1-qPCR-R | GCAATACGGAGCCAATCA | qRT-PCR |

**Table S3**

| Primers name | Primer sequence (5’-3’) | use |
| --- | --- | --- |
| VaflM-qPCR-F | GACTGTCAATGCCATCGCC | qRT-PCR |
| VaflM-qPCR-R | CGGTGACCTTGATAACTT | qRT-PCR |
| Vayg1-qPCR-F | GTTGCGACGAGTTCTTGT | qRT-PCR |
| Vayg1-qPCR-R | ACCATCACCTTGCCCATA | qRT-PCR |
| VDH1-qPCR-F | GTCTATTCATCTGGTTCCTCCCTA | qRT-PCR |
| VDH1-qPCR-R | CAAACCTCTTACAATGTTGACGC | qRT-PCR |

**Table S4**

| Primers name | Primer sequence (5’-3’) | use |
| --- | --- | --- |
| Vdβt-F | AACAACAGTCCGATGGATAATTC | qRT-PCR |
| Vdβt-R | GTACCGGGCTCGAGATCG | qRT-PCR |
| GhAct-F | CCTATGTTGCCCTGGACTATGAGC | qRT-PCR |
| GhAct-R | GGACAACGGAATCTCTCAGCTCC | qRT-PCR |

**Table S5**

| Primers name | Primer sequence (5’-3’) | use |
| --- | --- | --- |
| VDAG_04467 | F:ATCCGACTGCACTACATCGA | qRT-PCR |
|  | R:ATGGCGGACTGGCCCTTGAC |  |
| VDAG_01002 | F:GGACCAGAAGGACCCCAATG | qRT-PCR |
|  | R:ACAACCTGAATGTGGCTGGA |  |
| VDAG_04642 | F:AACGTCACCGTCGCCGCGGGC | qRT-PCR |
|  | R:CGCCGGCGTACCACGCGCGC |  |
| VDAG_08658 | F:CGTTCTCGAGGCTGAGAGCA | qRT-PCR |
|  | R:GGGCTTCGTGGCGTGGTTGA |  |
| VDAG_07154 | F:TGCCTGCCGACATGGAGCAT | qRT-PCR |
|  | R:AAGGTCGGTCGCTTTAACAA |  |
| VDAG_05792 | F:GTCATGAACAACACGCCCAT | qRT-PCR |
|  | R:GCGTCTTGAAGTGCAGCTGC |  |
| VDAG_03661 | F:TTCCAACAGCACCAGCAA | qRT-PCR |
|  | R:GCAATACGGAGCCAATCA |  |
| VDAG_01371 | F:CTGGTTTCTTTAGTCTGTTG | qRT-PCR |
|  | R:TTGACGCGCTTCTCAAACGA |  |
| VDAG_10388 | F:GGCCATTGCCATTCAGACGT | qRT-PCR |
|  | R:GTTCATCGGGACGCCGGT |  |
| VDAG_10381 | F:TCAGCTGCGCGGCAATATTC | qRT-PCR |
|  | R:CGAGTTGTCCATTGGAGGCG |  |

**Table S6**

| Primers name | Primer sequence (5’-3’) | use |
| --- | --- | --- |
| AD-VdCysp | F:CCATGGAGGCCAGTGAATTCgatgttcattcctgtcatca | Yeast |
|  | R:AGCTCGAGCTCGATGGATCCgctgggtgctgccacgacga |  |
| AD-VdTgc | F:CCATGGAGGCCAGTGAATTCggaatgagattatcagtgatcc | Yeast |
|  | R: AGCTCGAGCTCGATGGATCCgtttgtcaaatagatagtttc |  |
| AD-VdAOX | F:CCATGGAGGCCAGTGAATTCatgctctcagcaagaacatcc | Yeast |
|  | R:AGCTCGAGCTCGATGGATCCctagatgacctcagccctctc |  |
| AD-VdRad24 | F:CCATGGAGGCCAGTGAATTCatgggccaagaagatgccgt | Yeast |
|  | R:AGCTCGAGCTCGATGGATCCttactcggcggccttgggggcc |  |
| AD-VdExt2 | F:CCATGGAGGCCAGTGAATTCggccaggctttatttgactg | Yeast |
|  | R:AGCTCGAGCTCGATGGATCCttatatcgccaagataccct |  |
| BD-VdSti1 | F:TGGCCATGGAGGCCGAATTCatggctaccgcagacgagctca | Yeast |
|  | R:CGCTGCAGGTCGACGGATCCttaccgaccaacgcgaataa |  |
| nLUC-VdSti1 | F:CGGGGGACGAGCTCGGTACCatggctaccgcagacgagctca | LCI |
|  | R:GCAATACGGAGCCAATCAccgaccaacgcgaataacg |  |
| cLUC-VdExt2 | F:GTACGCGTCCCGGGGCGGTACCggccaggctttatttgactg | LCI |
|  | R:GAACGAAAGCTCTGCAGGTCGttatatcgccaagataccct |  |
